# Succinate Regulates Exercise‐Induced Muscle Remodelling by Boosting Satellite Cell Differentiation Through Succinate Receptor 1

**DOI:** 10.1002/jcsm.13670

**Published:** 2024-12-26

**Authors:** Yifan Shi, Da Zhou, Haoyang Wang, Longchang Huang, Xuejin Gao, Gulisudumu Maitiabula, Li Zhang, Xinying Wang

**Affiliations:** ^1^ Clinical Nutrition Service Center, Department of General Surgery, Nanjing Jinling Hospital, Affiliated Hospital of Medical School Nanjing University Nanjing Jiangsu China; ^2^ Department of Gastrointestinal Surgery Affiliated Hospital of Jiangnan University Wuxi Jiangsu China

**Keywords:** muscle remodelling, PKCη, satellite cells, succinate, SUCNR1

## Abstract

**Background:**

Skeletal muscle remodelling can cause clinically important changes in muscle phenotypes. Satellite cells (SCs) myogenic potential underlies the maintenance of muscle plasticity. Accumulating evidence shows the importance of succinate in muscle metabolism and function. However, whether succinate can affect SC function and subsequently coordinate muscle remodelling to exercise remains unexplored.

**Methods:**

A mouse model of high‐intensity interval training (HIIT) was used to investigate the effects of succinate on muscle remodelling and SC function by exercise capacity test and biochemical methods. Mice with succinate receptor 1 (SUCNR1)‐specific knockout in SCs were generated as an in vivo model to explore the underlying mechanisms. RNA sequencing of isolated SCs was performed to identify molecular changes responding to succinate‐SUCNR1 signalling. The effects of identified key molecules on the myogenic capacity of SCs were investigated using gain‐ and loss‐of‐function assays in vitro. To support the translational application, the clinical efficacy of succinate was explored in muscle‐wasting mice.

**Results:**

After 21 days of HIIT, mice supplemented with 1.5% succinate exhibited striking gains in grip strength (+0.38 ± 0.04 vs. 0.26 ± 0.03 N, *p* < 0.001) and endurance (+276.70 ± 55.80 vs. 201.70 ± 45.31 s, *p* < 0.05), accompanied by enhanced muscle hypertrophy and neuromuscular junction regeneration (*p* < 0.001). The myogenic capacity of SCs was significantly increased in gastrocnemius muscle of mice supplemented with 1% and 1.5% succinate (+16.48% vs. control, *p* = 0.008; +47.25% vs. control, *p* < 0.001, respectively). SUCNR1‐specific deletion in SCs abolished the modulatory influence of succinate on muscle adaptation in response to exercise, revealing that SCs respond to succinate–SUCNR1 signalling, thereby facilitating muscle remodelling. SUCNR1 signalling markedly upregulated genes associated with stem cell differentiation and phosphorylation pathways within SCs, of which p38α mitogen‐activated protein kinase (MAPK; fold change = 6.7, *p* < 0.001) and protein kinase C eta (PKCη; fold change = 12.5, *p* < 0.001) expressions were the most enriched, respectively. Mechanistically, succinate enhanced the myogenic capacity of isolated SCs by activating the SUCNR1–PKCη–p38α MAPK pathway. Finally, succinate promoted SC differentiation (1.5‐fold, *p* < 0.001), ameliorating dexamethasone‐induced muscle atrophy in mice (*p* < 0.001).

**Conclusions:**

Our findings reveal a novel function of succinate in enhancing SC myogenic capacity via SUCNR1, leading to enhanced muscle adaptation in response to exercise. These findings provide new insights for developing pharmacological strategies to overcome muscle atrophy–related diseases.

## 1 Introduction

Skeletal muscle is the largest organ, accounting for approximately 40% of body mass, and it contributes to whole‐body health by influencing metabolism and mobility. The clinical significance of skeletal muscle lies in its unparalleled plasticity manifested as specialized phenotypic remodelling in response to environmental cues [1]. Remodelling leading to an oxidative muscle fibre phenotype reduces the risk of metabolic diseases [2]. Muscle adaptations towards hypertrophic phenotypes and enhanced strength can offset the morbidity and mortality of muscle‐wasting disorders associated with age (sarcopenia) and cancer (cachexia) [3, 4]. Additionally, neural adaptation in muscle, an early paracrine response, improves muscle strength independent of hypertrophy [5]. Notably, physical exercise is a critical means of inducing changes in muscle remodelling, thus leading to exercise capacity as an effective therapy for preventing and treating multiple chronic diseases [6]. However, muscle adaptation to exercise is highly variable in animals and humans, with some individuals exhibiting stronger adaptive responses to the same exercise stimulations than others [7, 8]. The heterogeneity of muscle adaptive responses could be attributable to genetics and environmental factors, including advanced age and chronic disease [9, 10]. Therefore, developing pharmacological strategies that mimic or enhance the effects of exercise to improve muscle function is necessary.

Recently, our study utilizing meta‐omics analysis found that succinate was enriched in soldiers with superior exercise responses and positively correlated with physical phenotypes [11]. The positive impact of succinate on skeletal muscle physiology has recently gained attention. For instance, succinate restores oxygen consumption of skeletal muscle under mitochondrial dysfunction [12]. Long‐term treatment of cardiomyocytes with succinate could activate the hypertrophic signalling pathway [13]. Moreover, endogenous succinate secreted by muscle cells during exercise mediates muscle remodelling, including increased strength and fast‐twitch fibre phenotypes, through its cognate receptor succinate receptor 1 (SUCNR1) [14]. However, the extent to which dietary succinate initiates muscle adaptation in response to exercise remains unclear. Furthermore, the molecular mechanism through which succinate regulates fibre‐type remodelling remains elusive owing to the controversy regarding whether SUCNR1 is expressed in mature myotubes or nonmyofibrillar resident cells [14, 15].

Notably, a crucial driving force underlying robust muscle plasticity is muscle stem cells, called satellite cells (SCs) [16]. SCs are in a quiescent state below the basal lamina and adjacent to myofibers. Upon muscle injury or increased load, SCs are activated and undergo asymmetric division, wherein daughter cells self‐renew to repopulate the stem cell pool or continue towards myogenic lineage differentiation, eventually promoting muscle regeneration and hypertrophy [17]. Successful targeted muscle regeneration through SC interventions can effectively improve muscle function in various disease models, including sarcopenia and Duchenne muscular dystrophy [18, 19]. Furthermore, SCs also contribute to neuromuscular junction (NMJ) regeneration [20] that transfer information from motor neurons to muscles to generate movement. However, the effect of succinate on SC function has been poorly investigated.

In this study, we demonstrated that succinate further stimulated exercise‐induced muscle remodelling by promoting SC differentiation. Mechanistically, using genetic and pharmacological tools, we found that protein kinase C eta (PKCη) activation downstream of SUCNR1 agonism led to the phosphorylation of p38α mitogen‐activated protein kinase (MAPK) within SCs, which is both necessary and sufficient to enhance myogenic capacity of SCs. Our findings reveal a novel pharmacological function of succinate in muscle remodelling by targeting SCs, shedding light on developing promising therapeutic strategy for muscle‐wasting disorders.

## 2 Materials and Methods

### 2.1 Animals

Male C57BL/6 specific–pathogen‐free mice (8–10 weeks old) were purchased from Gempharmatech Co., Ltd. (Nanjing, China) and raised in the Model Animal Research Center of Nanjing University. Animals were kept in individual cages under ambient temperature (20°C–26°C), 40%–70% humidity, with a 12‐h light/dark cycle. Mice were unrestricted in activity, with free access to food and water. Animal experiments were approved by the Animal Care and Use Committee of Jinling Hospital (2021NZKY‐047‐01).

Mice with SUCNR1‐specific knockout (KO) in SCs were generated by crossing *Pax7‐CreER* and *Sucnr1* ^*fl/fl*^ mice. The genotype was *Pax7‐CreER* ^*+/−*^; *SUCNR1* ^*fl/fl*^ designated as homozygous (SUCNR1^SC/KO^) and *Pax7‐CreER* ^*−/−*^; *Sucnr1* ^*fl/fl*^ designated as control littermate (SUCNR1^SC/WT^). SC‐specific *SUCNR1* deletion was induced via intraperitoneal administration of tamoxifen (T5648, Sigma, St. Louis, MO, USA) dissolved in corn oil at 2 mg per 20 g body weight for seven consecutive days as previously described [21]. *Pax7‐CreER* mice were kindly provided by Dr Liwei Xie from the Institute of Microbiology, Guangdong Academy of Sciences, originally purchased from the Jackson Laboratory (Stock No. 017763), and *SUCNR1* ^*fl/fl*^ mice were directly purchased from Gempharmatech Co., Ltd. (Strain No. T052144).

### 2.2 Mouse Model of HIIT

Mice were acclimated to a motorized treadmill (KW‐PT, NJKEWBIO, Nanjing, China) for 5 days, running at 10 m/min for 15 min and then randomly divided into three groups (*n* = 10) with drinking water supplemented with 0%, 1% or 1.5% sodium succinate. Succinate‐containing water was freshly prepared and changed every 2 days. HIIT was performed based on a previously described method [22]. Briefly, HIIT involved treadmill running 4 days a week for 3 weeks. In each training session, the mice ran 15 bouts of 2 min at 100% of the maximal running speed interspersed by 2 min of active recovery running at 30% of the maximal running speed for a total of 60 min. The body weight and exercise capacity were measured before HIIT and 7, 14 and 21 days after the HIIT. The daily water consumption was recorded during the training period. Mice were euthanized after 21 days, and the whole blood, gastrocnemius (GA), tibialis anterior (TA) and extensor digitorum longus (EDL) were harvested for further processing.

### 2.3 Muscle Atrophy Mouse Model

SUCNR1^SC/WT^ and SUCNR1^SC/KO^ mice were randomly assigned to two groups (*n* = 10), respectively. All groups received a daily intraperitoneal injection of water‐soluble dexamethasone (Beyotime, Nanjing, China) at 10‐mg/kg body weight, with or without 1.5% succinate in drinking water. The body weight and exercise capacity were assessed before and 7, 14 and 21 days after the dexamethasone treatment. At the experimental endpoints (Day 22), mice were euthanized for further analyses.

### 2.4 Statistical Analyses

Statistical analyses were performed using GraphPad Prism 9 software. Experimental results are presented as mean ± standard deviation. Statistical significance was calculated using an unpaired Student's *t*‐test to compare the control and experimental groups. A one‐way or two‐way analysis of variance (ANOVA) with post hoc Bonferroni tests was used to compare more than two groups. Statistical significance was set at *p* < 0.05.

## 3 Results

### 3.1 Succinate Supplementation Increases Exercise Capacity

**FIGURE 1:**
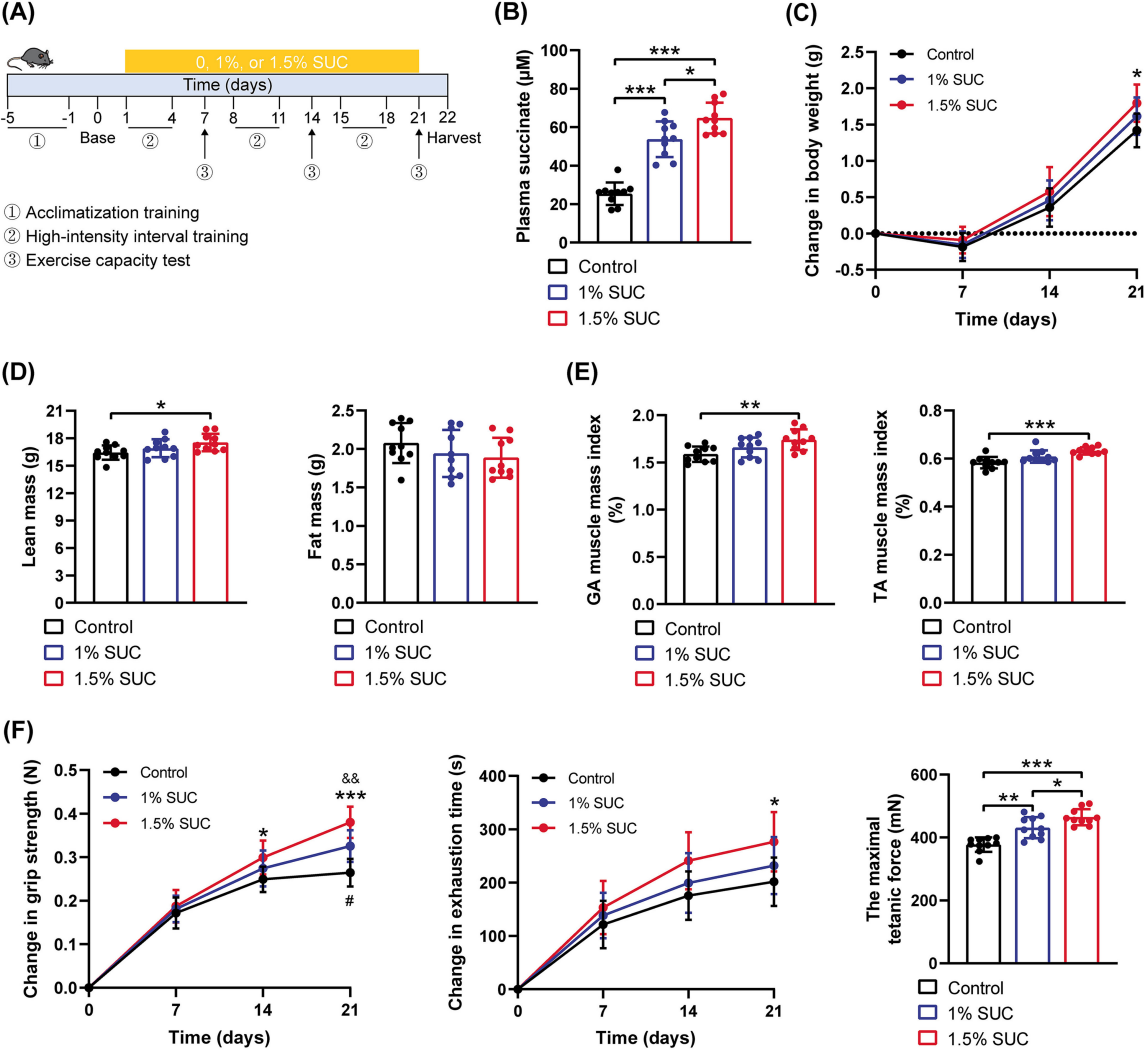
Exercise capacity increased in mice supplemented with succinate (SUC). (A) Timeline characterizing time points of SUC supplementation, high‐intensity interval training (HIIT), exercise capacity test and sample harvest. (B) Comparison of plasma SUC level among three groups following 21 days of HIIT. (C) Absolute changes in body weight from mice supplemented with 0%, 1% or 1.5% SUC during HIIT. Lean mass, fat mass (D), gastrocnemius (GA) and tibialis anterior (TA) mass indices (E) of mice supplemented with 0%, 1% or 1.5% SUC after completing the HIIT protocol. (F) Absolute changes in grip strength (left) and exhaustion time (middle) from mice supplemented with 0%, 1% or 1.5% SUC during the HIIT protocol. The maximal tetanic force in extensor digitorum longus of mice with 0%, 1% or 1.5% SUC after completing the HIIT protocol (right). Data are presented as mean ± SD. One‐way analysis of variance (ANOVA) with Bonferroni multiple comparisons (BMC) was employed in (B), (D), (E) and (F) (right). Two‐way ANOVA with BMC was employed in (C) and (F) (left and middle) where ^#^1% SUC vs. control, *1.5% SUC vs. control, and ^&^1.5% SUC vs. 1% SUC. *p or ^#^ *p* < 0.05, **p or ^&&^ *p* < 0.01, ***p < 0.001; *n* = 10 per group.

To characterize the effects of succinate on exercise capacity gains in response to HIIT, drinking water supplemented with 0%, 1% or 1.5% sodium succinate was provided to mice for 21 days (Figure 1A). The mice exhibited no aversion to succinate (Figure S1A), and succinate‐supplemented drinking water increased their succinate serum levels (Figure 1B). All sedentary mice displayed identical baseline characteristics (Figure S1B–D). Following a 3‐week HIIT protocol, mice supplemented with succinate exhibited significantly increased body weight gain, mainly contributed by an increase in lean mass (Figure 1C,D). A similar increase was observed in the GA and TA muscle mass, and their mass indices normalized by body weight following succinate supplementation (Figures 1E and S1E). Notably, succinate supplementation improved muscle contraction properties during HIIT in a concentration‐dependent manner, as indicated by increased grip strength (Figure 1F—left), exhaustion running time (Figure 1F—middle) and maximal tetanic force (Figure 1F—right). These results suggest that succinate enhances HIIT‐induced exercise capacity gain, accompanied by increased muscle mass.

### 3.2 Succinate Enhances Hypertrophic Muscle Adaptations

**FIGURE 2:**
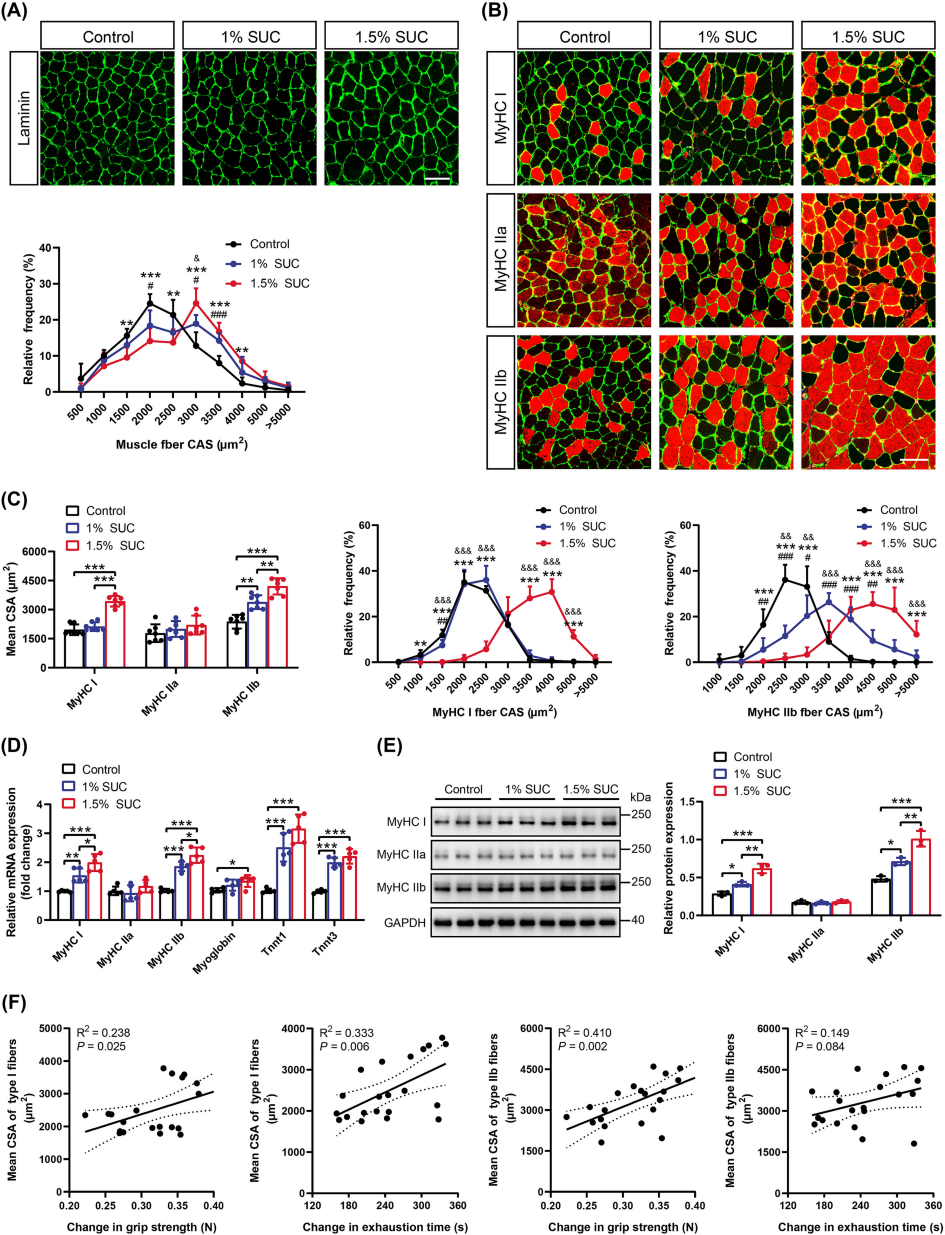
Succinate enhances hypertrophic adaptations in muscles. (A) Representative laminin staining (top) and frequency histogram (bottom) of cross‐sectional area (CSA) in gastrocnemius (GA) muscle harvested from mice supplemented with 0%, 1% or 1.5% succinate (SUC). Laminin, green; scale bars, 100 μm. (B) The representative images of immunofluorescent staining for laminin (green), myosin heavy chain (MyHC) I, MyHC IIa and MyHC IIb (red) in GA muscle of mice supplemented with 0%, 1% or 1.5% SUC. Scale bars, 100 μm. (C) The mean CSA analysis of MyHC I, MyHC IIa and MyHC IIb in GA muscles (left). Frequency histogram of MyHC I (middle) and MyHC IIb (right) CSA in GA muscles. (D) Real‐time quantitative PCR analyses of *MyHC I*, *MyHC IIa*, *MyHC IIb*, myoglobin, *TnnT1* and *TnnT3* mRNA levels in GA muscles from mice supplemented with 0%, 1% or 1.5% SUC. (E) Western blotting and semi‐quantitative analyses of MyHC I, MyHC IIa and MyHC IIb protein expression in GA muscle among three groups. (F) Pearson correlation analyses between changes in grip strength and exhaustion time (*x*‐axis) and the mean CSA of Type I and Type IIb fibres (*y*‐axis). Data are presented as mean ± SD. One‐way ANOVA with Bonferroni multiple comparisons (BMC) was employed in (A) and (C) (middle, right) where ^#^1% SUC vs. control, *1.5% SUC vs. control, and ^&^1.5% SUC vs. 1% SUC. One‐way ANOVA with BMC was employed in (C) (left), (D) and (E). *p, ^#^ *p* or ^&^ *p* < 0.05, **p, ^##^ *p* or ^&&^ *p* < 0.01, and ***p, ^###^ *p* or ^&&&^ *p* < 0.001; *n* = 7 per group (A, B, C and F), *n* = 5 per group (D), *n* = 3 per group (E).

To further determine the changes in specific myofiber morphology, we focused on the GA muscle, which contains an equal number of fast and slow‐twitch fibres [15]. GA cross‐sections were stained with laminin, demonstrating visual differences in myofiber size (Figure 2A—top). Notably, most myofibers in the 1% and 1.5% succinate groups had much larger CSAs than those in the control group (Figure 2A—bottom). Fibre‐type analysis revealed that succinate promoted increased CSA in oxidative (Type I) slow‐twitch and glycolytic (Type IIb) fast‐twitch fibres; however, no obvious change was observed in oxidative (Type IIa) fast‐twitch fibres (Figure 2B,C). Consistently, succinate markedly upregulated slow‐twitch fibre‐associated genes, *MyHC I*, myoglobin and troponin T1 (*TnnT1*), and fast‐twitch fibre‐associated genes, including *MyHC IIb* and *TnnT3* (Figure 2D). Western blotting of muscle protein lysates revealed increased MyHC I and MyHC IIb protein levels in the GA of mice that received succinate‐containing drinking water (Figure 2E). Furthermore, a positive correlation was observed between the mean CSA of Type I and Type IIb fibres, along with an increased exercise capacity during training (Figure 2F). Overall, these data demonstrate that succinate is a positive regulator of myofiber hypertrophy in response to HIIT, supporting succinate supplementation as a strategy to improve muscular hypertrophic adaptation.

### 3.3 Succinate Promotes Muscle Innervation

**FIGURE 3:**
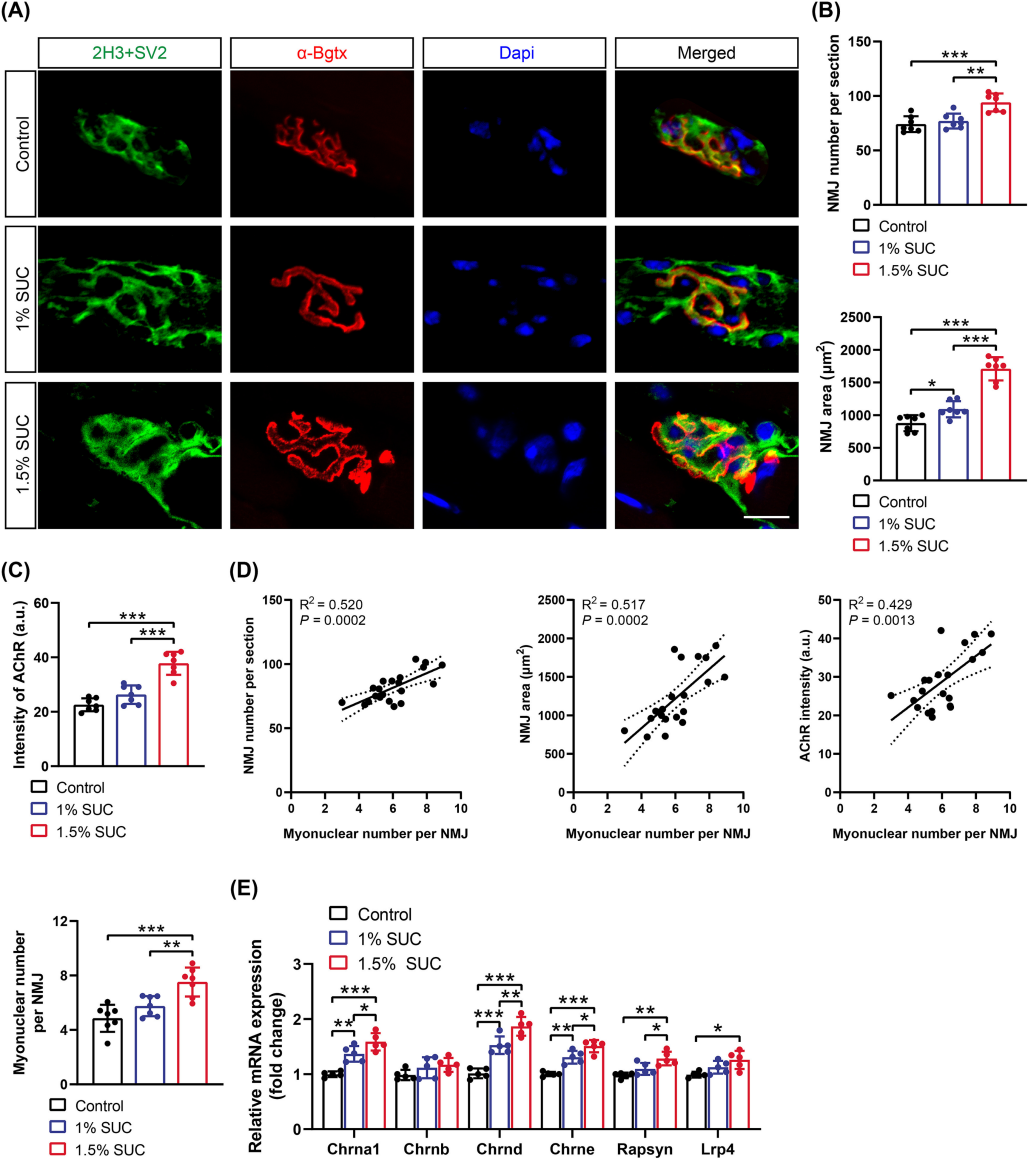
Succinate (SUC) promotes neuromuscular junction (NMJ) regeneration. (A) Maximal projections of confocal stacks of NMJs in gastrocnemius (GA) muscles from mice supplemented with 0%, 1% or 1.5% SUC. Muscle sections were stained with antibodies against neurofilament (2H3) and synaptic vesicle (SV2) (green) for presynaptic apparatus, and α‐bungarotoxin (α‐Bgtx) (red) for postsynaptic acetylcholine receptor (AChR). Scale bars, 20 μm. NMJ number, NMJ area (B), AChR intensity and myonuclear number per NMJ (C) in GA muscles of mice supplemented with 0%, 1% or 1.5% SUC. (D) Pearson correlation analyses for myonuclear number per NMJ and NMJ number (left), NMJ area (middle) and AChR intensity (right). (E) Real‐time quantitative PCR analysis of *Chrna1*, *Chrnb*, *Chrnd*, *Chrne*, *Rapsyn* and *Lrp4* mRNA levels in GA muscles from mice supplemented with 0%, 1% or 1.5% SUC. Data are presented as mean ± SD. One‐way ANOVA with Bonferroni multiple comparisons (BMC) was employed in (B), (C) and (E). *p < 0.05, **p < 0.01 and ***p < 0.001; *n* = 7 per group (A, B, C and D), *n* = 5 per group (E).

The central nervous system regulates skeletal muscle function through NMJs. To investigate the succinate‐induced modulation of neuronal adaptation in muscle, the morphology of NMJs in the GA muscle was evaluated using double immunostaining for the presynaptic and postsynaptic components. All GA muscles displayed consistent colocalization of the presynaptic neurofilament (2H3)/synaptic vesicle (SV2) and postsynaptic AChRs labelled with α‐Bgtx (Figure 3A). Notably, peripheral NMJs of the muscles from mice administered 1.5% succinate in drinking water were significantly altered, showing increased number, area, average AChR intensity and postsynaptic myonuclei content (Figure 3B,C). Postsynaptic myonuclei specialize in expressing genes associated with AChR assembly and NMJ regeneration [23]. Consistently, NMJ number, area and AChR intensity positively correlated with the postsynaptic myonuclear contents (Figure 3D). Moreover, we observed increased expression of genes encoding different acetylcholine receptor subunits (Chrn), including Chrna1, Chrnb, Chrnd and Chrne, in the GA muscles of mice fed succinate‐containing water, accompanied by the upregulation of genes associated with NMJ maturation and maintenance, including *Rapsyn* and low‐density lipoprotein receptor–related protein 4 (*Lrp4*) (Figure 3E). These results suggest that succinate can improve muscle innervation, which partly explains increased muscle strength.

### 3.4 Succinate Increases the Myogenic Capacity of SCs in Mice

**FIGURE 4:**
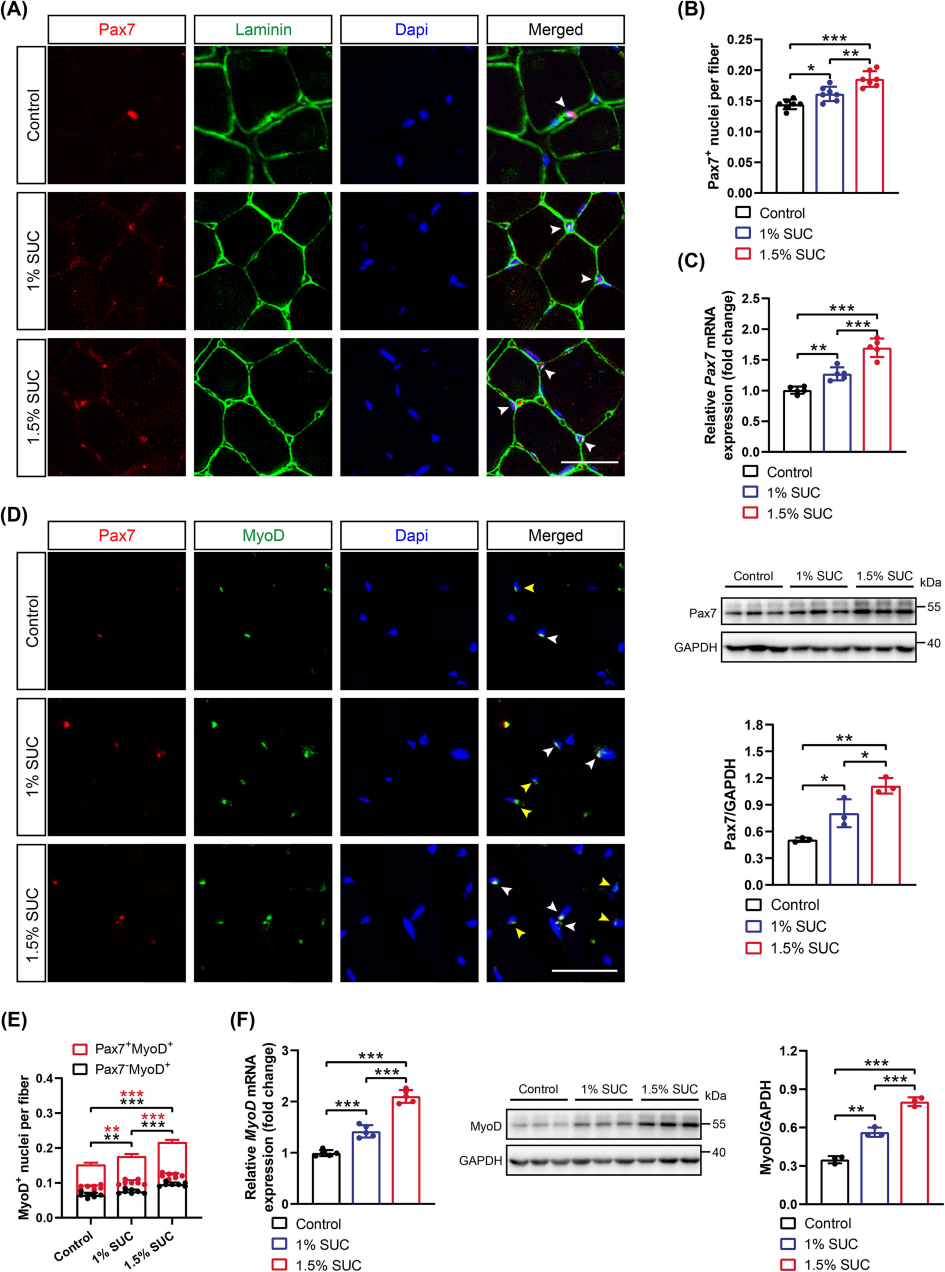
The myogenic capacity of satellite cells (SCs) increases in mice supplemented with succinate (SUC). (A) Representative images of immunofluorescent staining for Pax7 (red), laminin (green) and DAPI (blue) in gastrocnemius (GA) muscles from mice supplemented with 0%, 1% or 1.5% SUC. White arrows, PAX7^+^ nuclei. Scale bars, 20 μm. (B) The average number of PAX7^+^ SCs per GA section of mice supplemented with 0%, 1% or 1.5% SUC. (C) Real‐time quantitative PCR analysis of *Pax7* mRNA levels in GA muscles from mice supplemented with 0%, 1% or 1.5% SUC (top). Western blotting and semi‐quantitative analysis of PAX7 expression in GA muscles among three groups (bottom). (D) Representative images of immunofluorescent staining for Pax7 (red), MyoD (green) and DAPI (blue) in GA muscles from mice supplemented with 0%, 1% or 1.5% SUC. White arrows, *Pax7* ^*+*^ */MyoD* ^*+*^ nuclei; yellow arrows, *Pax7* ^*−*^ */MyoD* ^*+*^ nuclei. Scale bars, 20 μm. (E) The average number of *Pax7* ^*+*^ */MyoD* ^*+*^ and *Pax7* ^*−*^ */MyoD* ^*+*^ SCs per GA section of mice supplemented with 0%, 1% or 1.5% SUC. (F) Real‐time quantitative PCR analysis of *MyoD* mRNA levels in GA muscle from mice supplemented with 0, 1% or 1.5% SUC (left). Western blotting and semi‐quantitative analysis of MyoD protein expression in GA muscles among three groups (right). Data are presented as mean ± SD. *p < 0.05, **p < 0.01 and ***p < 0.001 by one‐way ANOVA with Bonferroni multiple comparisons (BMC); *n* = 7 per group (A, B, D and E), *n* = 5 per group (C [top], F [left]), *n* = 3 per group (C [bottom], F [right]).

SCs are crucial for muscle hypertrophy and neuronal adaptation; therefore, we explored whether succinate affects their function. First, we quantified the total number of SCs, as identified by PAX7‐positive staining, a canonical biomarker expressed explicitly in quiescent and activated SCs [24]. We observed that the number of *Pax7* ^*+*^ SCs was markedly increased in the GA muscles of mice supplemented with succinate than that in control mice (Figure 4A,B). PAX7 mRNA and protein levels were also significantly increased in mice that received succinate‐containing water (Figure 4C). Furthermore, as SC activation is marked by *MyoD* expression, *Pax7* ^*+*^ */MyoD* ^*+*^ and *Pax7* ^*−*^ */MyoD* ^*+*^ SCs represent SCs towards proliferation and differentiation, respectively [25]. The proliferation and differentiation of SCs were dramatically increased in the GA muscles of mice with succinate supplementation (Figure 4D,E). Succinate consistently induced *MyoD* mRNA and protein expression in a concentration‐dependent manner (Figure 4F). These data suggest that the increased myogenic capacity of SCs occurs with succinate supplementation, which may account for enhanced muscle regeneration and innervation.

### 3.5 SUCNR1 in SCs Mediates Succinate‐Induced Muscle Remodelling

Next, we investigated whether the G‐protein‐coupled receptor SUCNR1 mediated the aforementioned effects of succinate. A previous study indicated that SUCNR1 in myotubes induces fibre‐type remodelling [15]. However, in our study, although SUCNR1 was detected in the whole muscle (Figure S2A), it was not expressed in muscle fibres, exhibiting anticolocalization with the myofibrillar marker desmin (Figure S2B). Instead, SUCNR1 immunofluorescence correlated with SCs based on its strong colocalization with PAX7 (Figure S2B). To further corroborate this, we found that succinate had no direct stimulatory effect on C2C12 myoblast differentiation or the transcriptional regulation of myosin heavy chains (Figure S2C–E). Therefore, we hypothesized that SUCNR1 in SCs is essential for succinate‐induced muscle remodelling in response to exercise.

**FIGURE 5:**
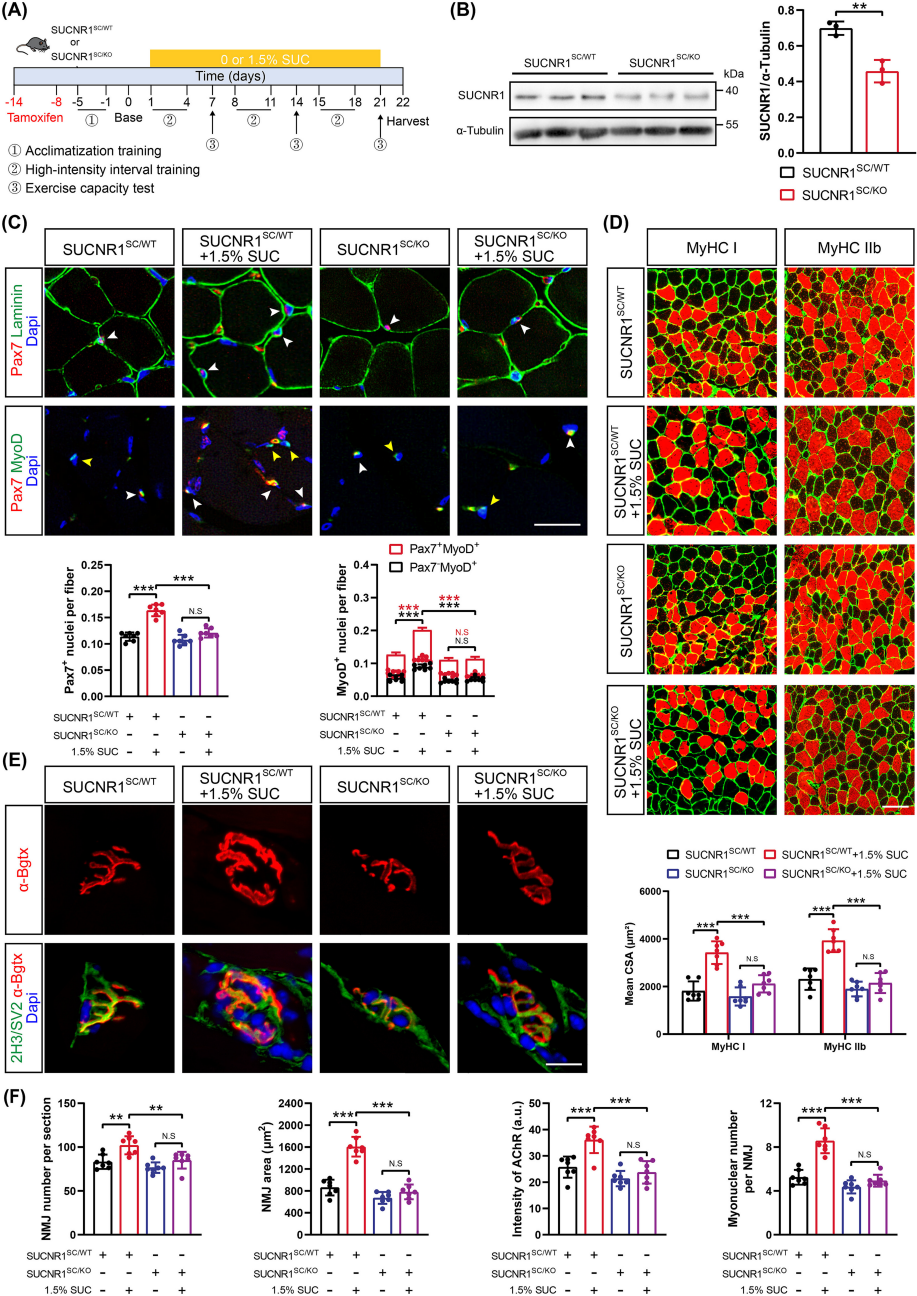
Succinate receptor 1 (SUCNR1)‐specific deletion in satellite cells (SCs) abolishes succinate‐induced muscle remodelling. (A) Timeline characterizing time points of tamoxifen injection, succinate (SUC) supplementation, high‐intensity interval training (HIIT), exercise capacity test and sample harvest. (B) Western blotting and semi‐quantitative analysis of SUCNR1 protein expression in gastrocnemius (GA) muscles from SUCNR1^SC/WT^ and SUCNR1^SC/KO^ mice. (C) Representative images of immunofluorescent staining for PAX7 (red), laminin, MyoD (green) and DAPI (blue) in GA muscles from SUCNR1^SC/WT^ and SUCNR1^SC/KO^ mice supplemented with or without 1.5% SUC (top). White arrows, *Pax7* ^*+*^ nuclei or *Pax7* ^*+*^ */MyoD* ^*+*^ nuclei; yellow arrows, *Pax7* ^*−*^ */MyoD* ^*+*^ nuclei. Scale bars, 20 μm. The average number of *Pax7* ^*+*^, *Pax7* ^*+*^ */MyoD* ^*+*^ and *Pax7* ^*−*^ */MyoD* ^*+*^ SCs per GA section among these four groups (bottom). (D) Representative images of immunofluorescent staining for laminin (green), myosin heavy chain (MyHC) I, and MyHC IIb (red) in GA muscles from SUCNR1^SC/WT^ and SUCNR1^SC/KO^ mice supplemented with or without 1.5% SUC (top). Scale bars, 100 μm. The semi‐quantitative analyses for mean cross‐sectional area (CSA) of MyHC I and MyHC IIb in GA muscles from these four groups (bottom). (E) Representative images of immunofluorescent staining for presynaptic neurofilament (2H3)/synaptic vesicle (SV2) (green) and postsynaptic acetylcholine receptors (AChRs) labelled with α‐bungarotoxin (α‐Bgtx) (red) in GA muscles from SUCNR1^SC/WT^ and SUCNR1^SC/KO^ mice supplemented with or without 1.5% SUC. Scale bars, 20 μm. (F) NMJ number, NMJ area, AChR intensity and myonuclear number per NMJ in GA muscles among these four groups. Data are presented as mean ± SD. Student's *t*‐test was employed in (B). One‐way ANOVA with Bonferroni multiple comparisons was employed in (C), (D) and (F). N.S., not significant, *p < 0.05, **p < 0.01 and ***p < 0.001; *n* = 10 per group (A), *n* = 3 per group (B), *n* = 7 per group (C, D, E and F).

To examine this, SC‐specific SUCNR1 KO mice were generated by intercrossing mice with *Pax7‐CreER* and *SUCNR1* ^*fl/fl*^ alleles. SUCNR1‐KO mice and their control littermates were designated SUCNR1^SC/KO^ and SUCNR1^SC/WT^, respectively. Nine days after intraperitoneal injection of tamoxifen, mice were separated into four groups, SUCNR1^SC/KO^ or SUCNR1^SC/WT^ with 0% or 1.5% succinate supplementation, and subsequently subjected to HIIT for 21 days (Figure 5A). Results indicated a significant decrease in SUCNR1 protein expression in the GA muscle of experimental mice (Figure 5B), and no difference in succinate‐containing water consumption was observed among the four groups during HIIT (Figure S3A). As expected, the number of total (*Pax7* ^*+*^), proliferation (*Pax7* ^*+*^ */MyoD* ^*+*^) and differentiation SCs (*Pax7* ^*−*^ */MyoD* ^*+*^) was remarkably reduced in the GA muscle of SUCNR1^SC/KO^ mice with 1.5% succinate compared with those in SUCNR1^SC/WT^ mice receiving 1.5% succinate, indicating that the succinate‐induced increased myogenic capacity of SCs was mediated by SUCNR1 (Figures 5C and S3B). Furthermore, SUCNR1‐specific deletion in SCs effectively abolished the regulatory effects of succinate on body weight (Figure S3C), muscle mass (Figure S3D) and exercise capacity (Figure S3E), including grip strength and exhaustion time. Consistent with these findings, succinate‐induced enhanced hypertrophic adaptation (Figures 5D and S3F) and NMJ regeneration (Figures 5E,F and S3G) were blocked in the GA muscles of SUCNR1^SC/KO^ mice. Collectively, these data suggest that SCs respond to succinate–SUCNR1 signalling to facilitate muscle remodelling.

### 3.6 Succinate–SUCNR1 Signalling Controls SC Differentiation Transcriptional Programs

**FIGURE 6:**
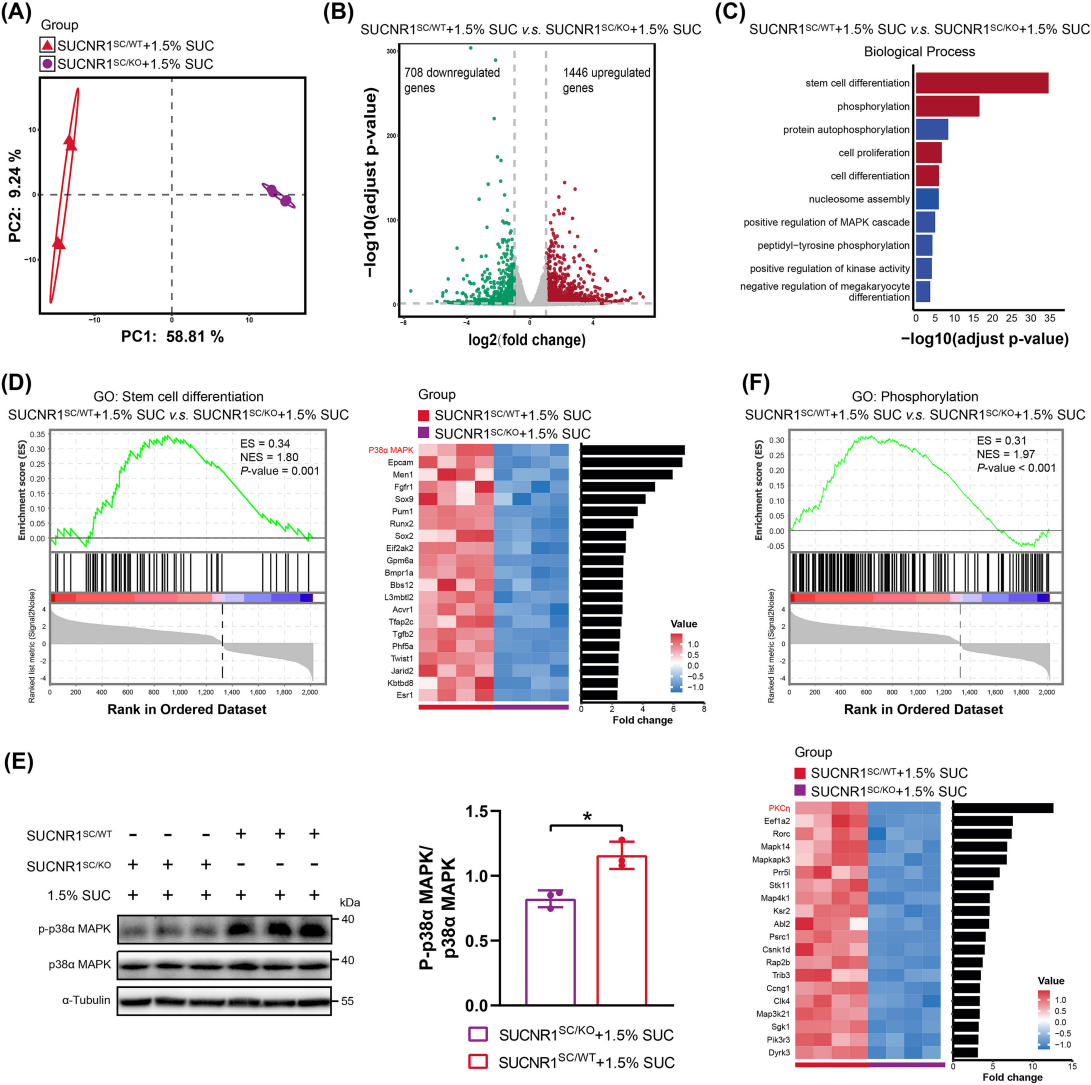
Succinate receptor 1 (SUCNR1) signalling regulates satellite cell (SC) differentiation transcriptional programs. (A) Principal component analysis (PCA) for the transcriptome of isolated SCs from SUCNR1^SC/WT^ and SUCNR1^SC/KO^ mice supplemented with 1.5% succinate (SUC). (B) Volcano plot displaying the adjusted *p*‐value (*y*‐axis) and fold change (*x*‐axis) of the identified genes in the transcriptomic data. The colour of the circle represents differentially expressed genes (DEGs) with upregulation (red) or downregulation (green) in SCs isolated from SUCNR1^SC/WT^ mice supplemented with 1.5% SUC. (C) Gene ontology (biological process) analysis for upregulated DEGs in SCs from SUCNR1^SC/WT^ mice supplemented with 1.5% SUC. (D) Gene set enrichment analysis (GSEA) analysis for stem cell differentiation pathway between SUCNR1^SC/WT^ and SUCNR1^SC/KO^ mice supplemented with 1.5% SUC (left). Heatmap and fold change of stem cell differentiation–related gene expression between the two groups (right). (E) Western blotting and semi‐quantitative analysis of phosphorylated p38α MAPK and p38α MAPK protein expression in SCs from SUCNR1^SC/WT^ and SUCNR1^SC/KO^ mice supplemented with 1.5% SUC. (F) GSEA analysis for phosphorylation pathway between SUCNR1^SC/WT^ and SUCNR1^SC/KO^ mice supplemented with 1.5% SUC (top). Heatmap and fold change of phosphorylation‐related gene expression between the two groups (bottom). Data are presented as mean ± SD. Student's *t*‐test was employed in E. *p < 0.01; *n* = 4 per group (A, B, C, D and F), *n* = 3 per group (E). Each *n* = SCs isolated from a single mouse.

To probe the molecular changes in SCs responding to SUCNR1‐mediated signalling, we performed RNA sequencing of freshly isolated SCs from SUCNR1^SC/WT^ and SUCNR1^SC/KO^ mice supplemented with 1.5% succinate that completed the HIIT protocol (Figure S4A–C). PCA revealed dissimilar molecular signatures between SUCNR1^SC/WT^ and SUCNR1^SC/KO^ mice supplemented with 1.5% succinate (Figure 6A). In total, 2154 DEGs with 1446 upregulated and 708 downregulated genes were identified between the two groups (Figure 6B). GO analysis (biological process) revealed that stem cell differentiation, phosphorylation, cell proliferation and differentiation were upregulated in the SCs of SUCNR1^SC/WT^ mice supplemented with 1.5% succinate (Figure 6C). Specifically, the enriched biological function of stem cell differentiation was further demonstrated using GSEA (Figure 6D—left). Among the top 20 DEGs associated with stem cell differentiation, p38α MAPK expression was the most enriched with a 6.7‐fold change (Figure 6D—right), and this was also observed for all p38 MAPK isoforms between the two groups (Figure S4D). Importantly, Western blotting confirmed that p38α MAPK phosphorylation was dramatically increased in SCs of SUCNR1^SC/WT^ mice supplemented with 1.5% succinate (Figure 6E). Correspondingly, this effect was accompanied by enriched biological phosphorylation and the upregulation of phosphorylation‐related genes, particularly *PKCη*, that exhibited the highest fold changes among the top 20 DEGs and detected PKC isoforms (Figures 6F and S4E). Overall, these data indicate that succinate–SUCNR1 signalling drives the transcriptional programs of SC differentiation, which may be mediated through the PKCη–p38α MAPK axis.

### 3.7 Succinate Promotes SC Differentiation Through the SUCNR1–PKCη–p38α MAPK Pathway

We investigated whether the PKCη–p38α MAPK axis is involved in succinate–SUCNR1 signalling in SCs. First, succinate‐induced PKC phosphorylation peaked at 30 min in the SCs isolated from C57BL/6 mice (Figure S5A). Among the significantly different conventional (PKCγ), novel (PKCδ, η) and atypical (PKCζ) PKC isoforms (Figure S4E), we observed that only PKCη translocated from the cytosol to membrane responding to the 30 min succinate treatment (Figure S5B). SUCNR1 siRNA (si‐SUCNR1) transfection in SCs abrogated succinate‐induced PKCη activation (Figure S5C). Furthermore, we examined the involvement of p38α MAPK in the succinate‐induced signalling pathway. The phosphorylation of p38α MAPK in SCs was detected between 60 and 90 min following treatment (Figure S5D). In addition, the succinate‐induced increase in the phosphorylation of p38α MAPK was significantly attenuated by SUCNR1 and PKCη knockdown (Figure S5E,F). These results suggest that succinate can activate the PKCη–p38α MAPK axis via SUCNR1 in SCs.

**FIGURE 7:**
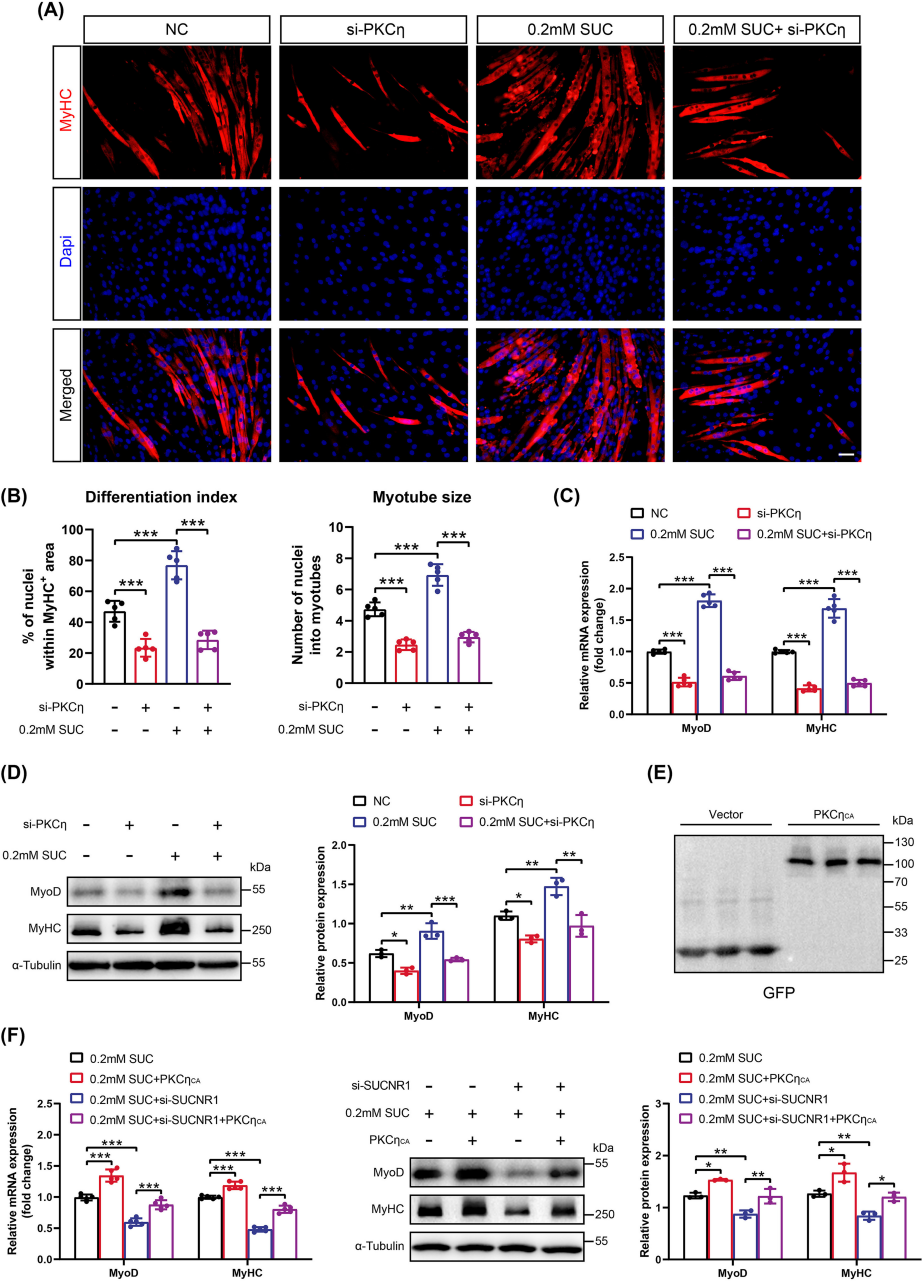
Succinate promotes satellite cell (SC) differentiation via activating succinate receptor 1 (SUCNR1)–protein kinase C eta (PKCη) pathway in vitro. (A) Representative images of immunofluorescence staining for myosin heavy chain (MyHC) (red) and DAPI (blue) in SCs transfected with PKCη small interfering RNA (si‐PKCη) and negative control (NC) after treatment with or without 0.2‐mM succinate (SUC) during differentiation. Scale bars, 50 μm. (B) Quantification of differentiation index and myotube size in si‐PKCη and NC transfected SCs after treatment with or without 0.2‐mM SUC during differentiation. (C) Real‐time quantitative PCR analyses of *MyoD* and *MyHC* mRNA levels in si‐PKCη and NC transfected SCs after treatment with or without 0.2‐mM SUC during differentiation. (D) Western blotting and semi‐quantitative analyses of MyoD and MyHC protein expression in si‐PKCη and NC transfected SCs after treatment with or without 0.2‐mM SUC during differentiation. (E) Green fluorescent protein (GFP) expression as determined by Western blotting in SCs transfected with empty plasmid (vector) and plasmid expressing constitutively active PKCη (PKCη_CA_). (F) Real‐time quantitative PCR analyses of *MyoD* and *MyHC* mRNA levels in SCs cotransfected with vector or PKCη_CA_ and SUCNR1 small interfering RNA (si‐SUCNR1) or NC after treatment with 0.2‐mM SUC during differentiation (left). Western blotting and semi‐quantitative analyses of MyoD and MyHC protein expression in SCs among the four groups (right). Data are presented as mean ± SD. *p < 0.05, **p < 0.01 and ***p < 0.001 by one‐way ANOVA with Bonferroni multiple comparisons; *n* = 5 per group (A, B, C and F [left]), *n* = 3 per group (D, E and F [right]).

We further hypothesized that the SUCNR1–PKCη–p38α MAPK pathway could be essential for promoting SC differentiation. To test this, isolated SCs were transfected with PKCη siRNA (si‐PKCη) or negative control (NC) and subsequently induced for differentiation. Remarkably, adding succinate to SCs resulted in robust concentration–dependent differentiation stimulation (Figure S6A,B) and the mRNA expression of myogenesis‐related genes, including *MyoD* and *MyHC* (Figure S6C). The si‐PKCη transfection in SCs markedly reduced the protein expression of PKCη (Figure S5F). SCs transfected with si‐PKCη were largely insensitive to succinate‐induced enhancement of myogenic differentiation, as illustrated by the decrease in differentiation index, myotube size (Figure 7A,B) and the mRNA and protein expression of myogenesis‐related genes (Figure 7C,D). To examine the sufficiency of PKCη activity to promote SC differentiation, we cotransfected plasmid expressing PKCη_CA_ or vector and si‐SUCNR1 or NC into SCs. Both PKCη_CA_ and vector were expressed in SCs (Figure 7E), and the expression of SUCNR1 in SCs was decreased by approximately fourfold compared to that in the NC group (Figure S6D). Notably, cotransfection with si‐SUCNR1 and vector significantly reduced the succinate‐induced myogenic marker expression in SCs, which was largely reversed by the expression of PKCη_CA_ (Figure 7F). Immunofluorescence staining of MyHC also indicated that PKCη_CA_ expression markedly promoted the differentiation of SCs transfected with si‐SUCNR1 and NC (Figure S6E,F).

To probe the role of p38α MAPK in the phenotypes of SCs mediated via the SUCNR1–PKCη pathway, isolated SCs were transfected with the vector and PKCη_CA_. We discovered that PKCη_CA_ expression recapitulated the enhanced myogenic differentiation of SCs previously observed with SUCNR1 activation, which could be effectively blocked by VX‐745, a p38α MAPK inhibitor (Figure S7A,B). RT‐qPCR and Western blotting further confirmed that the SC treatment with VX‐745 in the presence or absence of PKCη_CA_ substantially inhibited the myogenesis‐related gene expression (Figure S7C,D). Taken together, the SUCNR1–PKCη–p38α MAPK signalling pathway mediates the succinate‐induced myogenic differentiation of SCs.

### 3.8 Succinate Ameliorates Muscle Atrophy in Dexamethasone‐Treated Mice by Improving SC Myogenic Capacity

**FIGURE 8:**
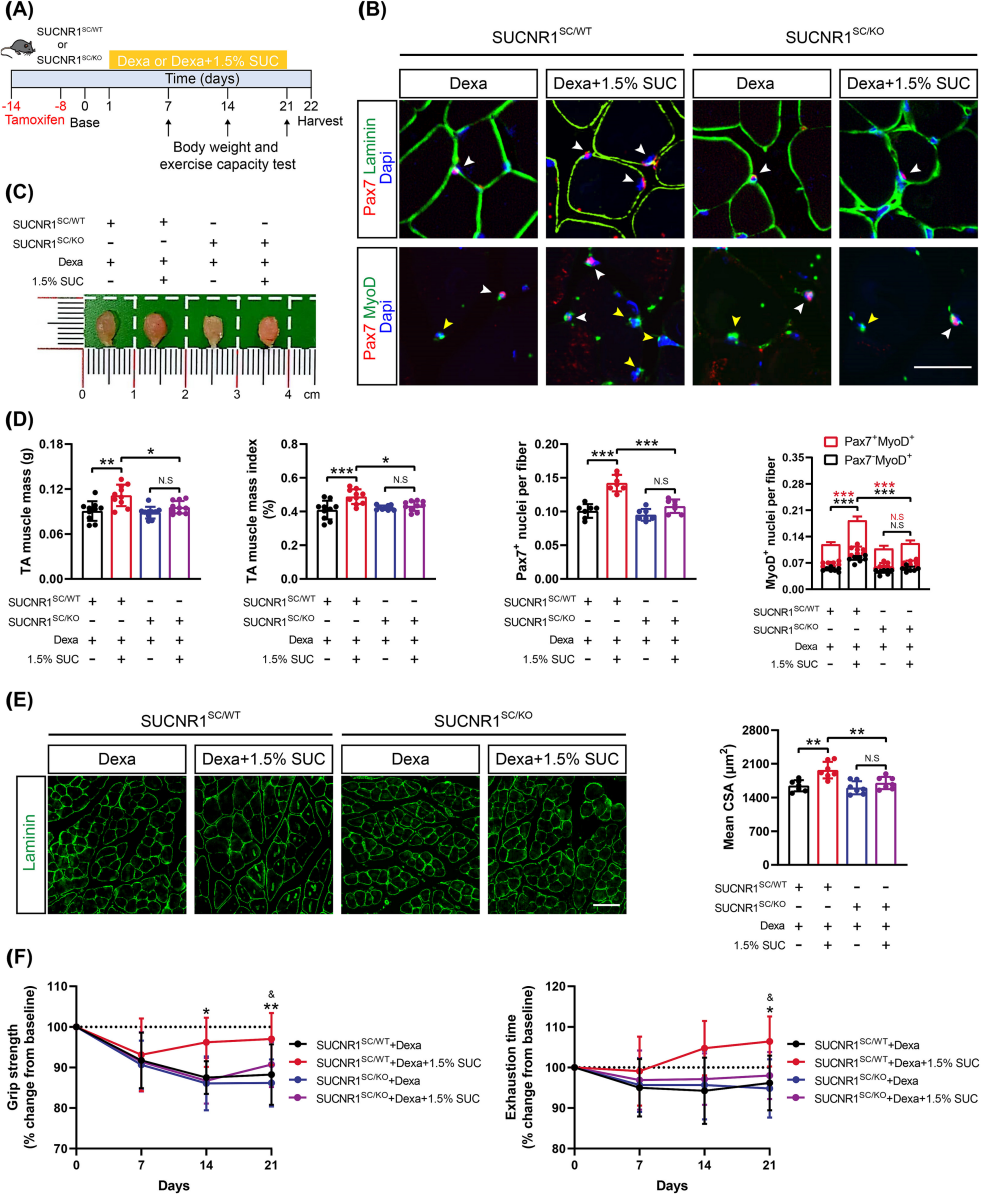
Succinate (SUC) ameliorates dexamethasone (Dexa)‐induced muscle atrophy in mice by enhancing the myogenic capacity of satellite cells (SCs). (A) Timeline characterizing time points of tamoxifen injection, Dexa injection, SUC supplementation, exercise capacity test and sample harvest. (B) Representative images of immunofluorescent staining for PAX7 (red), laminin, MyoD (green) and DAPI (blue) in tibialis anterior (TA) muscles from SUCNR1^SC/WT^ and SUCNR1^SC/KO^ mice injected by Dexa and supplemented with or without 1.5% SUC (top). White arrows, *Pax7* ^*+*^ nuclei or *Pax7* ^*+*^ */MyoD* ^*+*^ nuclei; yellow arrows, *Pax7* ^*−*^ */MyoD* ^*+*^ nuclei. Scale bars, 20 μm. The average number of *Pax7* ^*+*^, *Pax7* ^*+*^ */MyoD* ^*+*^ and *Pax7* ^*−*^ */MyoD* ^*+*^ SCs per TA section among the four groups (bottom). (C) Representative image of TA muscle from SUCNR1^SC/WT^ and SUCNR1^SC/KO^ mice injected with Dexa and supplemented with or without 1.5% SUC. (D) TA muscle mass and mass index in mice from the four groups. (E) Representative laminin staining (green) of cross‐sectional area (CSA) in TA muscles from SUCNR1^SC/WT^ and SUCNR1^SC/KO^ mice injected by Dexa and supplemented with or without 1.5% SUC (left). Scale bars, 50 μm. Semi‐quantitative analyses of mean CSA in TA muscles from the four groups (right). (F) Percentage change from baseline of grip strength (left) and exhaustion time (right) in mice. Data are presented as mean ± SD. One‐way ANOVA with Bonferroni multiple comparisons (BMC) was employed in (B), (D) and (E). Two‐way ANOVA with BMC was employed in (F) where *SUCNR1^SC/WT^ + Dexa + 1.5% SUC vs. SUCNR1^SC/WT^ + Dexa and ^&^SUCNR1^SC/WT^ + Dexa + 1.5% SUC vs. SUCNR1^SC/KO^ + Dexa + 1.5% SUC. N.S., not significant, *p or ^&^ *p* < 0.05, **p < 0.01, ***p < 0.001; *n* = 10 per group (A, C, D and F), *n* = 7 per group (B, E).

As succinate enhances muscle remodelling, it may hold therapeutic potential for muscle wasting, akin to the effects of exercise. To test this hypothesis, classical models of muscle atrophy were established in SUCNR1^SC/WT^ and SUCNR1^SC/KO^ mice by intraperitoneally injecting dexamethasone with or without 1.5% succinate supplementation (Figures 8A and S8A). As high‐dose dexamethasone induces significant atrophy of fast‐twitch glycolytic muscle, we focused on the TA muscle, which contains approximately 90% fast‐twitch glycolytic myofibers [26]. Compared with the other three groups, the number of total, proliferation and differentiation SCs was significantly increased in the TA muscle of SUCNR1^SC/WT^ mice injected with dexamethasone accompanied by succinate supplementation (Figure 8B). Consistent with the increased myogenic capacity of SCs, 3 weeks of succinate supplementation restored the decreased weight induced by dexamethasone to baseline in SUCNR1^SC/WT^ mice, whereas the other three groups maintained a 7%–10% body weight loss throughout the experiment due to reduced lean mass (Figure S8B–D). Specifically, simultaneous succinate supplementation during dexamethasone treatment counteracted glucocorticoid‐induced TA muscle wasting in SUCNR1^SC/WT^ mice, as supported by increased muscle mass (Figure 8C,D) and CSA (Figure 8E). Furthermore, SUCNR1^SC/WT^ mice supplemented with succinate exhibited a robust reversal of the dexamethasone‐induced decline in exercise capacity (Figures 8F and S8E). Notably, the beneficial effects of succinate in the muscle atrophy model were completely abolished in SUCNR1^SC/KO^ mice (Figure 8), emphasizing the indispensable role of SC activation mediated by succinate–SUCNR1 signalling. These findings demonstrate that succinate alleviates dexamethasone‐induced muscle atrophy in mice by promoting the myogenic capacity of SCs.

## 4 Discussion

In response to exercise, skeletal muscle adaptive remodelling can generate clinically significant changes in muscle phenotypes towards endurance or hypertrophic adaptations. However, muscle adaptations to physical exercise are highly heterogeneous in humans. In this study, we demonstrated a novel role for succinate as a critical regulator that improves SC myogenesis, thereby driving adaptive muscle remodelling in animals. Moreover, we provide mechanistic evidence that succinate modulates muscle phenotype by activating the SUCNR1–PKCη–p38α MAPK pathway within SCs (Figure S9). This discovery represents a significant advancement in the pharmacological regulation of the complex phenomenon of muscle remodelling.

Succinate has been considered a danger mediator in the immune system that boosts inflammatory responses [27]. Until recently, extracellular succinate reportedly regulates the metabolic function of tissues, exhibiting hormone‐like effects that include increasing insulin sensitivity and adipose thermogenesis [12]. Here, we identified the role of succinate as a positive regulator of increased strength, muscle hypertrophy and NMJ regeneration. Skeletal muscle consists of slow‐ and fast‐twitch fibre types with different contractile properties [28]. Slow‐twitch fibres (Type I) are rich in oxidative enzymes and specialized for continuous activity, whereas fast‐twitch fibres (Type II) are marked by glycolytic metabolism and specialized for phasic activity. Accordingly, succinate‐induced hypertrophy of Type I and IIb fibres could increase muscle mass and improve muscle strength and endurance, consistent with the exercise‐enhancing phenotypes observed in mice. Consistent with these findings, succinate secreted by skeletal muscles increases MyHC I and MyHC IIb protein levels [14]. In addition, peripheral motor neurons directly regulate muscle mobility and strength. The compromised interactions between motor nerves and targeted fibres lead to muscle atrophy and weakening (e.g., sarcopenia and spinal muscular atrophy) [29]. Conversely, restoring their connectivity and augmenting NMJ formation can increase muscle mass and strength after disease‐ or age‐induced denervation [30]. Therefore, the enhanced muscular innervation was at least partially responsible for the increased exercise capacity in mice supplemented with succinate in this study.

The mechanism underlying the succinate‐mediated effects in muscles remains unclear. Succinate is a ligand of SUCNR1, and the nature of SUCNR1‐dependent signalling cascades relies on target cell identity. Previous studies reported that SUCNR1 in muscle myotubes induces fibre‐type remodelling [15, 31]. However, we discovered that SUCNR1 is expressed in SCs rather than myofibers, consistent with the results reported by Reddy et al. [14], and it mediates the myogenic differentiation of SCs, which is vital for muscle hypertrophy. This discrepancy may be due to the cellular adaptation initiated by exercise training, resulting in SUCNR1 expression in different cell types. Moreover, activated SCs are the critical source of postsynaptic myonuclei that specifically express genes essential for NMJ assembly [32]. Consistent with vital roles, the enhanced SC myogenesis capacity induced by SUCNR1 agonism in this study was concomitant with increased postsynaptic myonuclei content, NMJ number and AChR intensity. Therefore, SUCNR1 in SCs mediates succinate‐induced muscle hypertrophy and NMJ regeneration. Supporting this hypothesis, SUCNR1‐specific deletion in SCs abolished the regulatory effects of succinate on muscle adaptation in mice. Accordingly, our findings demonstrate that SCs respond to succinate–SUCNR1 signalling to enhance hypertrophic remodelling and muscle innervation. However, the molecular changes in SCs mediated by SUCNR1 signalling require further investigation.

SUCNR1 agonism can trigger numerous intracellular phosphorylation cascades, including PKA, PKC and extracellular signal–regulated kinase pathways [12]. Here, we revealed that PKCη is downstream of SUCNR1 activation in SCs. Although SUCNR1‐induced phosphorylation of PKC has been previously reported [33, 34], none of these studies focus on the PKCη isoform and elucidate the function of the SUCNR1–PKCη axis in SCs. Our findings suggest that SUCNR1‐dependent activation of PKCη is necessary and sufficient to promote the enhanced myogenic differentiation of SCs. Consistent with our findings, PKCη also regulates the proliferation and differentiation of other cell types. For example, the PKCη pathway promotes the differentiation of endothelial cells derived from embryonic stem cells [35]. Under stress conditions, PKCη functions as a molecular switch that promotes the proliferation‐to‐differentiation transition in keratinocytes by upregulating *p27Kip1* mRNA.^S1^ Interestingly, other PKC subtypes, such as PKCδ, similarly control the phenotypic fate of multiple stem cells, including haematopoietic and mesenchymal stem cells.^S2^ ^,^ ^S3^ Therefore, our study suggested a novel function of the SUCNR1–PKCη axis in positively regulating SC differentiation.

Following demonstrating the role of PKCη in transducing succinate‐induced signalling, we further identified p38α MAPK as the downstream key molecule of PKCη activation during SC differentiation. Previously, p38α/β MAPK was considered a molecular switch to activate quiescent SCs.^S4^ Specifically, during the asymmetric division of SCs, the p38α/β MAPK pathway was activated in only one daughter cell in a PKCλ‐dependent manner, which induced *MyoD* expression and subsequently permitted cell myogenic progression [17].^S5^ In our study, the p38α MAPK inhibitor abolished the PKCη_CA_–induced enhanced myogenic differentiation of SCs, decreasing *MyoD* expression. Consistent with our findings, another study showed that p38α‐specific knockout in SCs resulted in impaired myogenic differentiation.^S6^ Therefore, succinate–SUCNR1 signalling induced the PKCη‐mediated phosphorylation of p38α MAPK in SCs, thereby stimulating the transcription of *MyoD* for myogenic differentiation.

Skeletal muscle loss is a pathological condition commonly observed in numerous chronic diseases and during aging, leading to devastating impacts on quality of life and the likelihood of death.^S7^ Muscle atrophy frequently correlates with muscle regeneration defects resulting from impaired SC activity and myogenesis.^S8^ Successful restoration of SC myogenic capacity effectively ameliorates muscle atrophy in mouse models of aging, sarcopenia and Duchenne muscular dystrophy [36].^S9^ ^,^ ^S10^ Consistently, our study demonstrated the beneficial effects of succinate on muscle atrophy induced by dexamethasone via enhancing SC myogenic capacity, suggesting that succinate may be a promising pharmaceutical molecule for the drug development in muscle atrophy–related diseases, such as sarcopenia and cachexia. However, to support the translational applicability of our study, the clinical efficacy of succinate should be further extended to human primary SCs.

This study has some limitations. First, we primarily focused on succinate–SUCNR1 signalling in SCs for muscle adaptation. Given a previous report on SUCNR1 expression in other nonmyofibrillar cell types, including stromal and endothelial cells [14], whether these cell populations respond to dietary succinate to coordinate SC function or muscle remodelling requires further exploration. Second, we discovered that succinate promoted the hypertrophic adaptation of Type I and Type IIb myofibers instead of Type IIa myofibers and NMJ regeneration by enhancing SC differentiation. However, the molecular mechanisms underlying the directed migration of SCs remain unexplored, and the number of differentiating SCs associated with specific fibre types and NMJs requires further quantification. Third, we did not perform an in vivo experiment to validate the effect of PKCη and p38α MAPK on SC function and muscle adaptation, although the isolated primary SCs were used for in vitro experiments. Finally, the clinical relevance of succinate in improving muscle function requires an in‐depth exploration in additional mouse models of aging, sarcopenia and cachexia for clinical translation.

Conclusively, our investigation uncovered a novel function of succinate in promoting the myogenic potential of SCs via SUCNR1 signalling, which can enhance muscle adaptation in response to exercise. This study reveals the potential of succinate as an exercise stimulus for individuals who strive to maintain their fitness and improve their performance. Furthermore, considering the impaired muscular adaptations in aging and chronic disease conditions, understanding the pharmacological effects of succinate that enhance clinically important adaptive remodelling in muscle will be invaluable for developing strategies to improve muscle function in specific populations.

## Conflicts of Interest

The authors declare no conflicts of interest.

## Supporting information


**Figure S1.** The baseline characteristics and the effects of succinate (SUC) on muscle mass. (A) Water consumption during high‐intensity interval training (HIIT). Body weight (B), grip strength (C) and exhaustion time (D) of mice at baseline. (E) Gastrocnemius (GA) and tibialis anterior (TA) muscle mass of mice supplemented with 0%, 1% or 1.5% SUC upon completion of the HIIT. Data are presented as mean ± SD. *p < 0.05, **p < 0.01 and ***p < 0.001 by one‐way ANOVA with Bonferroni multiple comparisons; *n* = 10 per group.
**Figure S2.** The cellular localization of succinate receptor 1 (SUCNR1) in gastrocnemius (GA) muscle. (A) Western blotting and semi‐quantitative analysis of SUCNR1 protein expression in GA muscle from mice supplemented with 0%, 1% or 1.5% succinate (SUC). (B) Representative images of immunofluorescent staining for SUCNR1 (red), Pax7 (yellow) and desmin (green) in GA muscles of mice (left). Scale bars, 40 μm. Manders’ colocalization coefficient among SUCNR1, Pax7 and desmin (right). Coefficients of less than 0.3 are considered completely anticolocalized, as validated by the anticolocalization control (distinct cell type markers). (C) Representative images of immunofluorescence staining for myosin heavy chain (MyHC) (red) and DAPI (blue) in C2C12 myoblasts after SUC treatment during differentiation. Scale bars, 50 μm. (D) Quantification of differentiation index and myotube size in C2C12 myoblasts after SUC treatment during differentiation. (E) Real‐time quantitative PCR analyses of *MyoD* and *MyHC* mRNA levels in C2C12 myoblasts after SUC treatment during differentiation. Data are presented as mean ± SD. *p < 0.05, **p < 0.01 and ***p < 0.001 by one‐way ANOVA with Bonferroni multiple comparisons; *n* = 3 per group (A, B), *n* = 5 per group (C, D, and E).
**Figure S3.** Succinate receptor 1 (SUCNR1) in SCs mediates succinate‐induced muscle adaption. (A) Water consumption during high‐intensity interval training (HIIT). (B) Real‐time quantitative PCR analyses of *PAX7* and *MyoD* mRNA levels in gastrocnemius (GA) muscles from SUCNR1^SC/WT^ and SUCNR1^SC/KO^ mice supplemented with or without 1.5% succinate (SUC) (left). PAX7 and MyoD protein expression as determined by Western blotting in GA muscles from the four groups (right). (C) Baseline body weight (left) and absolute changes in body weight (right) from SUCNR1^SC/WT^ and SUCNR1^SC/KO^ mice supplemented with or without 1.5% SUC during HIIT. (D) Lean mass, fat mass, gastrocnemius mass and gastrocnemius mass index from SUCNR1^SC/WT^ and SUCNR1^SC/KO^ mice supplemented with or without 1.5% SUC after completion of HIIT. (E) Baseline grip strength and exhaustion time from SUCNR1^SC/WT^ and SUCNR1^SC/KO^ mice (left). Absolute changes in grip strength and exhaustion time from SUCNR1^SC/WT^ and SUCNR1^SC/KO^ mice supplemented with or without 1.5% SUC during HIIT (right). (F) Real‐time quantitative PCR analyses of myosin heavy chain (*MyHC*) *I* and *MyHC IIb* mRNA levels in GA muscles from SUCNR1^SC/WT^ and SUCNR1^SC/KO^ mice supplemented with or without 1.5% SUC (top). MyHC I and MyHC IIb protein expression as determined by Western blotting in GA muscles from the four groups (bottom). (G) Real‐time quantitative PCR analysis of *Chrna1*, *Chrnb*, *Chrnd*, *Chrne*, *Rapsyn* and *Lrp4* mRNA levels in GA muscles from SUCNR1^SC/WT^ and SUCNR1^SC/KO^ mice supplemented with or without 1.5% SUC. Data are presented as mean ± SD. One‐way ANOVA with Bonferroni multiple comparisons (BMC) was employed in (A), (B), (C) (left), (D), (E) (left), (F) and (G). Two‐way ANOVA with BMC was employed in (C) (right) and (E) (right) where *SUCNR1^SC/WT^ + 1.5% SUC vs. SUCNR1^SC/WT^ and ^&^SUCNR1^SC/WT^ + 1.5% SUC vs. SUCNR1^SC/KO^ + 1.5% SUC. N.S., not significant, *p or ^&^ *p* < 0.05, **p or ^&&^ *p* < 0.01, and ***p or ^&&&^ *p* < 0.001; *n* = 10 per group (A, C, D and E), *n* = 5 per group (B [left], F [top] and G), *n* = 3 per group (B [right], F [bottom]).
**Figure S4.** Satellite cell (SC) isolation and RNA sequencing. (A) Representative fluorescence‐activated cell sorting (FACS) plots of SCs isolated from SUCNR1^SC/WT^ mice supplemented with 1.5% succinate (SUC) after completing the high‐intensity interval training (HIIT). (B) Representative FACS plots of SCs isolated from SUCNR1^SC/KO^ mice supplemented with 1.5% SUC after completing the HIIT. (C) Representative images of immunofluorescent staining for Pax7 (red) and DAPI (blue) in purified SC populations. (D) The gene expression levels of p38α, β, γ and δ mitogen‐activated protein kinase (MAPK) quantified based on the fragments per kilobase of transcript sequence per millions (FPKM) between SUCNR1^SC/WT^ and SUCNR1^SC/KO^ mice supplemented with 1.5% SUC. (E) Gene expression levels of conventional, novel and atypical protein kinase C (PKC) isoforms quantified using the FPKM between SUCNR1^SC/WT^ and SUCNR1^SC/KO^ mice supplemented with 1.5% SUC. Negative binomial exact test with Benjamini–Hochberg correction was employed in (D) and (E). *p < 0.05, **p < 0.01, and ***p < 0.001.
**Figure S5.** Succinate activates the protein kinase Cη (PKCη)–p38α mitogen‐activated protein kinase (MAPK) pathway through succinate receptor 1 (SUCNR1) in satellite cells (SCs). (A) Western blotting and semi‐quantitative analyses of phosphorylated PKC (p‐PKC) and PKC protein expression in isolated SCs treated with 0.1‐mM succinate (SUC) for different time periods (0–90 min). (B) Western blotting and semi‐quantitative analyses of PKC isoform protein expression in the membrane and cytoplasm of SCs treated with 0.1‐mM SUC for 30 min. (C) Western blotting and semi‐quantitative analyses of phosphorylated PKCη (p‐PKCη), PKCη and SUCNR1 protein expression in SCs transfected with SUCNR1 small interfering RNA (si‐SUCNR1) and negative control (NC) after treatment with or without 0.1‐mM SUC for 30 min. (D) Western blotting and semi‐quantitative analyses of phosphorylated p38α MAPK (p‐p38α MAPK) and p38α MAPK protein expression in isolated SCs treated with 0.1‐mM SUC for different time periods (0–90 min). (E) Western blotting and semi‐quantitative analyses of p‐p38α MAPK and p38α MAPK protein expression in SCs transfected with si‐SUCNR1 and NC after treatment with or without 0.1‐mM SUC for 60 min. (F) Western blotting and semi‐quantitative analyses of p‐p38α MAPK, p38α MAPK and PKCη protein expression in SCs transfected with si‐PKCη and NC after treatment with or without 0.1‐mM SUC for 60 min. Data are presented as mean ± SD. One‐way ANOVA with Bonferroni multiple comparisons was employed in (A), (C), (D), (E) and (F). Student’s *t*‐test was employed in (B). *p < 0.05, **p < 0.01 and ***p < 0.001; *n* = 3 per group.
**Figure S6.** Succinate enhances the myogenic differentiation of satellite cells (SCs) by succinate receptor 1 (SUCNR1)–protein kinase Cη (PKCη) activation. (A) Representative images of immunofluorescence staining for myosin heavy chain (MyHC) (red) and DAPI (blue) in isolated SCs following succinate (SUC) treatment during differentiation. Scale bars, 50 μm. (B) Quantification of differentiation index and myotube size in SCs following SUC treatment during differentiation. (C) Real‐time quantitative PCR analyses of *MyoD* and *MyHC* mRNA levels in SCs following SUC treatment during differentiation. (D) Western blotting and semi‐quantitative analysis of SUCNR1 protein expression in SCs transfected with SUCNR1 small interfering RNA (si‐SUCNR1) and negative control (NC). (E) Representative images of immunofluorescence staining for MyHC (red) and DAPI (blue) in SCs cotransfected with empty plasmid (vector) or plasmid expressing constitutively active PKCη (PKCη_CA_) and si‐SUCNR1 or NC after treatment with 0.2‐mM SUC during differentiation. Scale bars, 50 μm. (F) Quantification of differentiation index and myotube size in SCs cotransfected with vector or PKCη_CA_ and si‐SUCNR1 or NC after treatment with 0.2‐mM SUC during differentiation. Data are presented as mean ± SD. One‐way ANOVA with Bonferroni multiple comparisons was employed in (B), (C) and (F). Student’s *t*‐test was employed in (D). N.S., not significant, *p < 0.05, **p < 0.01, and ***p < 0.001; *n* = 5 per group (A, B, C, E and F), *n* = 3 per group (D).
**Figure S7.** The effects of p38α mitogen‐activated protein kinase (MAPK) on protein kinase Cη (PKCη)–induced satellite cell (SC) differentiation. (A) Representative images of immunofluorescence staining for myosin heavy chain (MyHC) (red) and DAPI (blue) in SCs transfected with empty plasmid (vector) and plasmid expressing constitutively active PKCη (PKCη_CA_) after treatment with or without VX‐745 during differentiation. Scale bars, 50 μm. (B) Quantification of differentiation index and myotube size in SCs transfected with vector and PKCη_CA_ after treatment with or without VX‐745 during differentiation. (C) Real‐time quantitative PCR analyses of *MyoD* and *MyHC* mRNA levels in SCs transfected with vector and PKCη_CA_ after treatment with or without VX‐745 during differentiation. (D) Western blotting and semi‐quantitative analyses of MyoD and MyHC protein expression in SCs transfected with vector and PKCη_CA_ after treatment with or without VX‐745 during differentiation. Data are presented as mean ± SD. *p < 0.05, **p < 0.01 and ***p < 0.001 by one‐way ANOVA with Bonferroni multiple comparisons; *n* = 5 per group (A, B and C), *n* = 3 per group (D).
**Figure S8.** The impact of succinate (SUC) on skeletal muscle in dexamethasone (Dexa)‐treated mice. (A) Water consumption during Dexa treatment. (B) Baseline body weight of mice. (C) Percentage change from baseline of body weight in mice. (D) Lean mass and fat mass of SUCNR1^SC/WT^ and SUCNR1^SC/KO^ mice injected by Dexa and supplemented with or without 1.5% SUC. (E) Grip strength and exhaustion time of mice at baseline. Data are presented as mean ± SD. One‐way ANOVA with Bonferroni multiple comparisons (BMC) was employed in (A), (B), (D) and (E). Two‐way ANOVA with BMC was employed in (C) where *SUCNR1^SC/WT^ + Dexa + 1.5% SUC vs. SUCNR1^SC/WT^ + Dexa and ^&^SUCNR1^SC/WT^ + Dexa + 1.5% SUC vs. SUCNR1^SC/KO^ + Dexa + 1.5% SUC. N.S., not significant, *p or ^&^ *p* < 0.05, **p < 0.01; *n* = 10 per group.
**Figure S9.** The schematic diagram depicting succinate in modulating muscle adaptive remodelling. Succinate supplementation promotes the myogenic differentiation of satellite cells through activating SUCNR1–PKCη–p38α MAPK pathway, which drives muscle adaptive remodelling, including enhanced muscle hypertrophy and innervation.
**Table S1.** Antibodies used for immunofluorescence (IF) or Western blotting (WB).
**Table S2.** Primer sequences.

## Data Availability

RNA‐sequencing data are available from the corresponding author upon reasonable request.
