# Supplementary material for: Succinate Regulates Exercise‐Induced Muscle Remodelling by Boosting Satellite Cell Differentiation Through Succinate Receptor 1
Source: J Cachexia Sarcopenia Muscle. 2024 Dec 26;16(1):e13670. doi: 10.1002/jcsm.13670 (PMC11670172; doi:10.1002/jcsm.13670)
Supplement: Supplementary file 1 — Figure S1. The baseline characteristics and the effects of succinate (SUC) on muscle mass. (A) Water consumption during high‐intensity interval training (HIIT). Body weight (B), grip strength (C) and exhaustion time (D) of mice at baseline. (E) Gastrocnemius (GA) and tibialis anterior (TA) muscle mass of mice supplemented with 0%, 1% or 1.5% SUC upon completion of the HIIT. Data are presented as mean ± SD. *p < 0.05, **p < 0.01 and ***p < 0.001 by one‐way ANOVA with Bonferroni multiple comparisons; n = 10 per group. Figure S2. The cellular localization of succinate receptor 1 (SUCNR1) in gastrocnemius (GA) muscle. (A) Western blotting and semi‐quantitative analysis of SUCNR1 protein expression in GA muscle from mice supplemented with 0%, 1% or 1.5% succinate (SUC). (B) Representative images of immunofluorescent staining for SUCNR1 (red), Pax7 (yellow) and desmin (green) in GA muscles of mice (left). Scale bars, 40 μm. Manders’ colocalization coefficient among SUCNR1, Pax7 and desmin (right). Coefficients of less than 0.3 are considered completely anticolocalized, as validated by the anticolocalization control (distinct cell type markers). (C) Representative images of immunofluorescence staining for myosin heavy chain (MyHC) (red) and DAPI (blue) in C2C12 myoblasts after SUC treatment during differentiation. Scale bars, 50 μm. (D) Quantification of differentiation index and myotube size in C2C12 myoblasts after SUC treatment during differentiation. (E) Real‐time quantitative PCR analyses of MyoD and MyHC mRNA levels in C2C12 myoblasts after SUC treatment during differentiation. Data are presented as mean ± SD. *p < 0.05, **p < 0.01 and ***p < 0.001 by one‐way ANOVA with Bonferroni multiple comparisons; n = 3 per group (A, B), n = 5 per group (C, D, and E). Figure S3. Succinate receptor 1 (SUCNR1) in SCs mediates succinate‐induced muscle adaption. (A) Water consumption during high‐intensity interval training (HIIT). (B) Real‐time quantitative PCR analyses of PAX7 and [file JCSM-16-e13670-s001.docx]

**Online Supplement**

**Succinate regulates exercise-induced muscle remodeling by boosting satellite cell differentiation through succinate receptor 1**

Yifan Shi^1,2†^, Da Zhou^1†^, Haoyang Wang^1^, Longchang Huang^1^, Xuejin Gao^1^, Gulisudumu Maitiabula^1^, Li Zhang^1^, Xinying Wang^1*^

^1^Department of General Surgery, Nanjing Jinling Hospital, Affiliated Hospital of Medical School, Nanjing University, Nanjing, Jiangsu, China.

^2^Department of Gastrointestinal Surgery, Affiliated Hospital of Jiangnan University, Wuxi, Jiangsu, China.

*Correspondence: wangxinying@nju.edu.cn (X.Y.W.)

**Supplemental Methods**

*Exercise capacity*

To assess improvements in exercise capacity, muscle strength and exercise endurance were evaluated. First, the maximum grip strength of the mice was measured thrice using a force meter (KW-ZL, NJKEWBIO, Nanjing, China), and the mean maximum force was used for the analysis. Furthermore, a treadmill running test was performed at an initial speed of 10 m/min, and speed was then ramped up 1 m/min every 2 min until exhaustion, defined as the point where the mice remained on the shockers that encouraged running for >10 s.^37^ Time to exhaustion was recorded, and the maximal running speed was identified as the final completed stage during the acceleration test. Mouse body composition was determined using a nuclear magnetic resonance system (NM21-060H-I, NIUMAG, Suzhou, China). All tests were performed in a blinded manner.

*Muscle force generation assay*

A muscle-contraction test was performed on the EDL muscles using a 3-in-1 whole animal system (1300A; Aurora Scientific, ON, Canada) as described previously.^36^ Mice were anesthetized with isoflurane throughout the procedure. The EDL was dissected, adjusted to the optimal length, and attached to a force transducer. The electrodes were placed in the EDL, and the specific tetanic force was recorded at 170 Hz using dynamic muscle control software (Aurora Scientific).

*C2C12 culture and differentiation*

Mouse C2C12 myoblasts obtained from the American Type Culture Collection were cultured in growth media, comprising Dulbecco’s Modified Eagle’s medium (DMEM; Gibco, CA, USA), 10% fetal bovine serum (FBS), and 1% penicillin/streptomycin, at 37 °C and 5% CO_2_. After reaching 80–90% confluence, myoblasts were induced with a differentiation medium containing DMEM with 2% horse serum (HS) for myogenic differentiation. Succinate (0.05, 0.1, and 0.2 mM) was co-cultured with C2C12 cells for 5 days from differentiation, and the medium containing succinate was changed every 2 days.

*SCs isolation*

SCs were isolated as described previously.^38^ TA and GA muscles were dissected, minced, and digested with 700 U/mL collagenase II (Merck KGaA, Darmstadt, Germany) at 37 °C for 60 min. Samples were subsequently centrifuged at 500 × *g* for 5 min, and the supernatant was incubated with 100 U/mL collagenase II and 1 U/mL dispase (Merck KGaA) at 37 °C for 30 min. The cell suspension was filtered through a 40-µm strainer and subsequently stained with the following antibodies purchased from Biolegend (San Diego, CA, USA): PE/cyanine7 anti-mouse CD31 (Cat#102418), APC anti-mouse CD45 (Cat#103112), anti-fluorescein anti-mouse Vcam1 (Cat#105706), and Alexa Fluor® 700 anti-mouse Sca-1 (Cat#108142) at 4 °C for 20 min. After washing off the unbound antibodies, the SCs were sorted by gating with CD31^-^CD45^-^Sca1^-^Vcam^+^ using a CytoFLEX flow cytometer (Beckman Coulter, Inc., CA, USA), and the results were analyzed using the FlowJo software (V10.9).

*SC culturing and differentiation*

Freshly isolated SCs were seeded at 8000 cells per well into a 6-well plate and maintained in growth media containing Ham's F10, 20% FBS, 1% penicillin/streptomycin, and 2.5 ng/mL fibroblast growth factor (Cellverse Bioscience Technology Co., Ltd., Shanghai, China) at 37 °C and 5% CO_2_. For myogenic differentiation, SC-derived primary myoblasts were induced with differentiation media (DMEM with 5% HS and 1% penicillin/streptomycin) for 5 days and then subjected to further experiments and analyses. Depending on the experimental condition, vehicle (PBS), succinate (0.05, 0.1, and 0.2 mM), or a p38α MAPK inhibitor (VX-745, 10 nM, MedChemExpress, Monmouth Junction, NJ, USA) were added to the differentiation media, and the culture medium was replaced every 2 days.

*RNA sequencing and analysis*

SCs were isolated from SUCNR1^SC/WT^ and SUCNR1^SC/KO^ mice supplemented with 1.5% succinate, which completed the HIIT protocol described above. Total RNA was extracted using a RNeasy Kit (74104; Qiagen, Hilden, Germany) and purified using an RNAClean XP Kit (A63987, Beckman Coulter). RNA concentration was measured using the NanoDrop Lite spectrophotometer (Thermo Fisher Scientific, Waltham, MA, USA), and RNA integrity was determined with 4200TapeStation (Agilent Technologies, Palo Alto, CA, USA). Libraries were constructed using the U-mRNAseq Library Prep Kit (AT4221, BAITAI-BIO, Shanghai, China). The libraries were then subjected to paired-end 150-bp sequencing using an Illumina NovaSeq 6000 (Illumina, San Diego, CA, USA).

Raw RNA-sequencing reads were trimmed using Fastp (v.0.20.0) and aligned to the mouse ENSEMBL genome using HISAT2 (v.2.1.0). The unique gene hit counts were summarized using the featureCounts module of the Subread package (v.1.5.2). All RNAs were quantified as fragments per kilobase million mapped reads using HTSeq (http://htseq.readthedocs.io/en/release_0.9.1). Principal component analysis (PCA) was employed to assess the differences in mRNA expression profiles in SCs between the two groups using the stats package in R version 4.3.0. Differentially expressed genes (DEGs), defined as |log2 fold change| >1 and adjusted *P*-value <0.05, were identified using edgeR. Gene ontology (GO) analysis was performed using the clusterProfiler package in R (http://www.r-project.org/) for upregulated DEGs (log_2_ fold change >1 and adjusted *P*-value <0.05). DEGs were further analyzed for biological processes using Gene Set Enrichment Analysis (GSEA), and the expression profiles of DEGs associated with altered pathways are shown as heatmaps generated by the pheatmap package of R.

*Small interfering RNA (siRNA) and plasmid transfection in SCs*

Freshly isolated SCs from C57BL/6 mice were seeded into 12-well plates at a density of 50,000 cells/well. Next, 5 μL of SUCNR1 (20 μM, GenePharma Co., Ltd., Shanghai, China) and PKCη siRNA (20 μM, GenePharma Co., Ltd.) were mixed with 200 μL Opti-MEM media (Thermo Fisher Scientific), respectively. Furthermore, 15 μL of RNAi-Mate (GenePharma Co., Ltd.) was added dropwise to the siRNA mixture and incubated for 10 min at 20 °C to form transfection complexes. These final solutions were added to the 12-well plate when the SCs reached 70–80% confluency. pCMV/MCS/EGFP/Neo plasmid expressing constitutively active PKCη (PKCη_CA_) was generated by GenePharma Co., Ltd. via site-directed mutagenesis of the pseudosubstrate region (A161E).^39^ SCs at approximately 80% confluency were co-transfected with 1.5 μg of empty plasmid (vector) or plasmid expressing PKCη_CA_ and SUCNR1 siRNA (100 nM) or NC in the 12-well plate. The media was replaced with the differentiation medium after 24 h. Cells were used 48 h later. The siRNA sequences for inhibiting SUCNR1 and PKCη were 5′-GCUCUUGCUCACUGUCAUUTT-3′ and 5′-CAGGAUGAGUUUAGAAACUTT-3,’ respectively.

*Immunofluorescence staining*

Fresh GA muscles were collected and immediately frozen using liquid nitrogen-cooled isopentane in an optimum cutting temperature compound. Next, 10-μm-thick transverse and longitudinal serial sections were prepared using a cryostat (CM1950; Leica, Wetzlar, Germany). For immunofluorescence staining, muscle sections or cells were fixed using 4% paraformaldehyde in PBS for 15 min, permeabilized with 0.1% Triton X-100 for 10 min, and immersed in 3% hydrogen peroxide for 10 min, followed by a 60-min block in blocking buffer containing 3% bovine serum albumin. Subsequently, samples were incubated overnight with the chosen primary antibodies at 4 °C. The antibodies used for immunofluorescence are listed in Table S1.

After washing with PBS, the slides were incubated for 60 min at 20 °C with appropriate secondary antibodies, including goat anti-rabbit immunoglobulin G (IgG)/Alexa Fluor 647 (A-21245, Invitrogen, Waltham, MA, USA), goat anti-mouse IgG/Alexa Fluor 488 (A-11001, Invitrogen), and goat anti-mouse IgM/Alexa Fluor 555 (bs-0368G-AF555, Bioss, Woburn, MA, USA). For NMJ staining, some sections were also incubated with CY5-conjugated alpha-bungarotoxin (α-Bgtx) (5 μg/mL; 90-2002-01, Biotium Inc., Shanghai, China) to label post-synaptic acetylcholine receptors (AChRs). To establish the cellular localization of SUCNR1, the sections were incubated with a horseradish peroxidase-conjugated secondary antibody (GB23303, Servicebio, Wuhan, China) at 20 °C for 50 min in the dark, and the corresponding antigens were individually incubated with the reagents of the tyramide signal amplification kits (G1223, G1231, and G1222, Servicebio) at 20 °C for 10 min. Before visualizing the next antigen, primary and secondary antibodies were eluted using an ethylenediaminetetraacetic acid antigen repair resolution (G1203, Servicebio). Nuclei were counterstained with DAPI (F6057, Sigma). Images were captured using a Pannoramic Scan System (3DHistech, Budapest, Hungary) or an LSM 880 laser confocal microscope (Zeiss, Oberkochen, Germany).

*Image analysis*

Two independent investigators blindly performed the imaging and morphometric measurements. Digital images were analyzed using the ImageJ software (v1.52a, NIH, Bethesda, MD, USA).

Myofiber cross-sectional areas (CSAs) were quantified in a single transversal section per muscle obtained from its mid-belly and immunostained with laminin or different subtype-specific anti-muscle myosin heavy chain (MyHC) antibodies. Five fields with approximately 150 myofibers per field were examined in each section. The results were illustrated as a frequency histogram. The mean CSA of isoform-specific myofibers (I, IIa, and IIb) in each mouse was calculated.

NMJ morphology was evaluated in longitudinal sections of the muscles. Z-stack optical sections (1.4-μm thick) were obtained using a confocal microscope (LSM880, Zeiss), and maximum intensity projections of stacks were created to reconstruct NMJs with the microscope software. NMJs with a clear overlap between pre- and post-synaptic labeling were defined as innervation. Five sections were examined for each muscle, in which the total number of NMJs was manually counted and approximately 50 NMJs from different randomly selected fields were assessed for morphometric parameters and immunofluorescence intensity. NMJ size was determined by manually outlining the post-synaptic area labeled with α-Bgtx. Post-synaptic myonuclei were identified based on >25% DAPI covered by an α-Bgtx-labeled post-synaptic apparatus.^20^

To assess SC content and activation status, sections were stained using PAX7 in conjunction with laminin or MyoD, and >200 myofibers per field were included in the analyses. Those loci co-stained with PAX7^+^ and DAPI^+^ within the laminin border were counted as the total number of SCs. The activation status of SCs was assessed based on the colocalization of PAX7, MyoD, and DAPI, and proliferating and differentiating SCs were labeled PAX7^+^/MyoD^+^ and PAX7^-^/MyoD^+^, respectively.^25^

*Protein isolation and western blotting*

Muscle tissues and cells were homogenized in radioimmunoprecipitation assay lysis buffer containing protease and phosphatase inhibitors (Thermo Fisher Scientific). The homogenate was incubated on ice for 30 min and subsequently centrifuged at 15,000 rpm for 15 min at 4 °C. The resulting supernatant was retained as the total protein lysate. To evaluate PKCη activity in the culture cells, membranous proteins and cytosolic fractions were extracted using an extraction kit (P0033; Beyotime) following the manufacturer's instructions. Protein concentrations were determined using a bicinchoninic acid assay kit (23227, Thermo Fisher Scientific). Equal amounts of protein (30 μg) from each sample were electrophoresed on 5–10% SDS-PAGE gels, transferred onto polyvinylidene fluoride membranes, and blotted with primary antibodies. The antibodies used are listed in Table S1. Images were acquired using a Tanon 4600SF Imaging System (Shanghai, China), and semi-quantitative analyses were performed using the ImageJ software.

*RNA extraction and Real-time quantitative PCR (RT-qPCR)*

Total RNA was extracted from the GA muscle and cells using TRIzol reagent (Invitrogen) and reverse-transcribed into cDNA using PrimeScript RT Master Mix (Takara Bio, Inc., Tokyo, Japan) following the manufacturer's instructions. RT-qPCR was performed using the SYBR qPCR Master Mix (Takara Bio, Inc.) in a ViiATM 7 qPCR System (Applied Biosystems, Foster City, CA, USA). The target mRNA expression relative to the *GAPDH* transcript level was calculated using the 2^−ΔΔCT^ method.^40^ Table S2 presents the primer sequences for gene amplification.

**Supplemental References**

37. Henriquez-Olguin C, Renani LB, Arab-Ceschia L, Raun SH, Bhatia A, Li Z, et al. Adaptations to high-intensity interval training in skeletal muscle require NADPH oxidase 2. Redox Biol 2019;**24**:101188.

38. Liu L, Cheung TH, Charville GW, Rando TA. Isolation of skeletal muscle stem cells by fluorescence-activated cell sorting. Nat Protoc 2015;**10**:1612-1624.

39. Suzuki T, Elias BC, Seth A, Shen L, Turner JR, Giorgianni F, et al. PKC eta regulates occludin phosphorylation and epithelial tight junction integrity. Proc Natl Acad Sci U S A 2009;**106**:61-66.

40. Livak KJ, Schmittgen TD. Analysis of relative gene expression data using real-time quantitative PCR and the 2(-Delta Delta C(T)) Method. Methods 2001;**25**:402-408.

S1. Hara T, Miyazaki M, Hakuno F, Takahashi S, Chida K. PKCeta promotes a proliferation to differentiation switch in keratinocytes via upregulation of p27Kip1 mRNA through suppression of JNK/c-Jun signaling under stress conditions. *Cell Death Dis* 2011;**2**:e157.

S2. Binder V, Li W, Faisal M, Oyman K, Calkins DL, Shaffer J, et al. Microenvironmental control of hematopoietic stem cell fate via CXCL8 and protein kinase C. *Cell Rep* 2023;**42**:112528.

S3. Datta A, Sarmah D, Kaur H, Chaudhary A, Mounica KL, Kalia K, et al. Post-stroke impairment of the Blood-Brain barrier and perifocal vasogenic edema is alleviated by endovascular mesenchymal stem cell administration: Modulation of the PKCdelta/MMP9/AQP4-Mediated pathway. *Mol Neurobiol* 2022;**59**:2758-2775.

S4. Jones NC, Tyner KJ, Nibarger L, Stanley HM, Cornelison DD, Fedorov YV, et al. The p38alpha/beta MAPK functions as a molecular switch to activate the quiescent satellite cell. *J Cell Biol* 2005;**169**:105-116.

S5. Troy A, Cadwallader AB, Fedorov Y, Tyner K, Tanaka KK, Olwin BB. Coordination of satellite cell activation and self-renewal by Par-complex-dependent asymmetric activation of p38alpha/beta MAPK. *Cell Stem Cell* 2012;**11**:541-553.

S6. Brien P, Pugazhendhi D, Woodhouse S, Oxley D, Pell JM. P38alpha MAPK regulates adult muscle stem cell fate by restricting progenitor proliferation during postnatal growth and repair. *Stem Cells* 2013;**31**:1597-1610.

S7. Henrot P, Blervaque L, Dupin I, Zysman M, Esteves P, Gouzi F, et al. Cellular interplay in skeletal muscle regeneration and wasting: Insights from animal models. *J Cachexia Sarcopenia Muscle* 2023;**14**:745-757.

S8. Bodine SC, Sinha I, Sweeney HL. Mechanisms of skeletal muscle atrophy and molecular circuitry of stem cell fate in skeletal muscle regeneration and aging. *J Gerontol A Biol Sci Med Sci* 2023;**78**:14-18.

S9. Price FD, von Maltzahn J, Bentzinger CF, Dumont NA, Yin H, Chang NC, et al. Inhibition of JAK-STAT signaling stimulates adult satellite cell function. *Nat Med* 2014;**20**:1174-1181.

S10.Rodriguez-Outeirino L, Hernandez-Torres F, Ramirez DAF, Rastrojo A, Creus C, Carvajal A, et al. MiR-106b is a novel target to promote muscle regeneration and restore satellite stem cell function in injured Duchenne dystrophic muscle. *Mol Ther Nucleic Acids* 2022;**29**:769-786.

**Supplemental Figures**

**
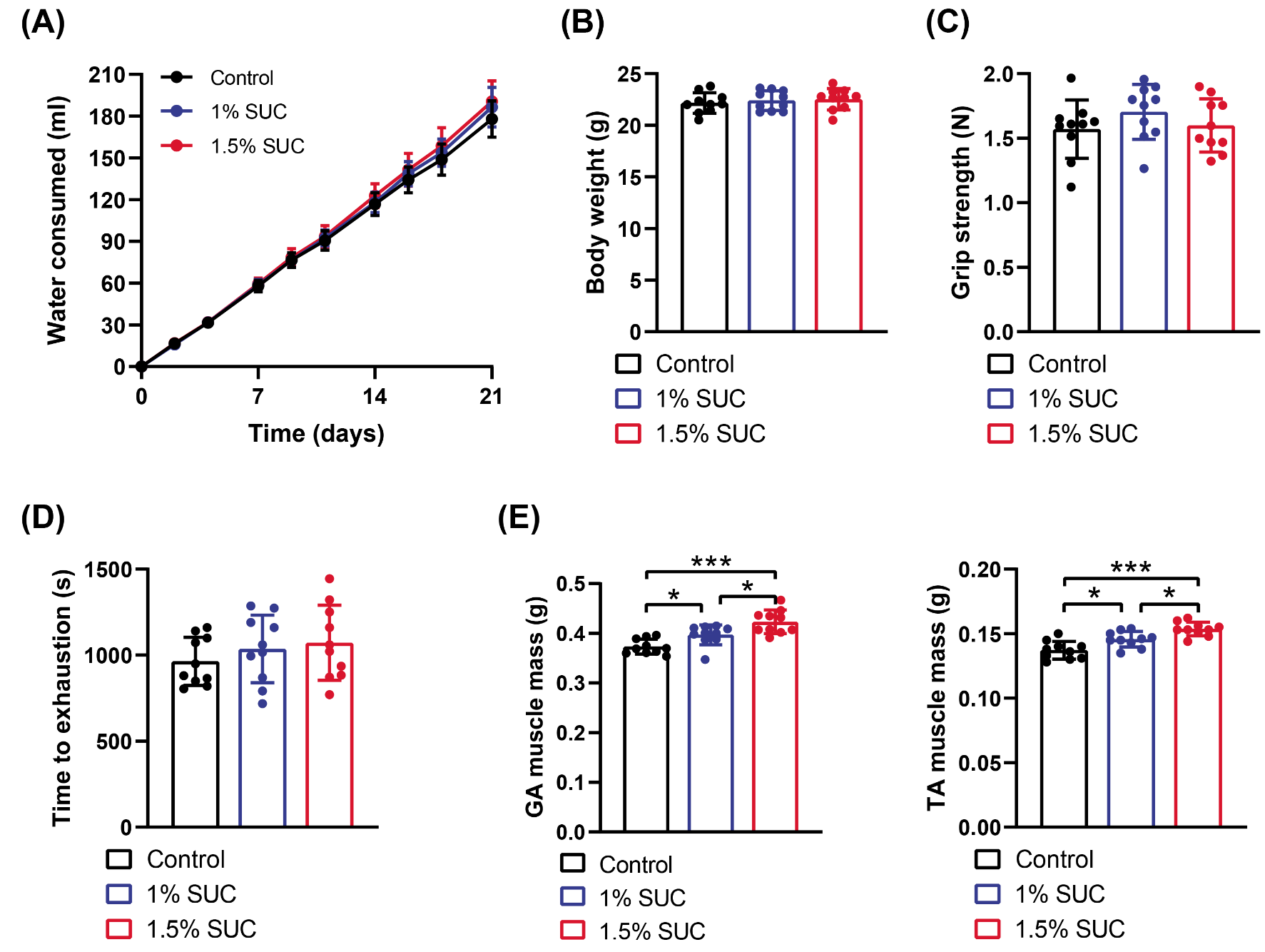
**

**Figure S1** The baseline characteristics and the effects of succinate (SUC) on muscle mass. (A) Water consumption during high-intensity interval training (HIIT). Body weight (B), grip strength (C), and exhaustion time (D) of mice at baseline. (E) Gastrocnemius (GA) and tibialis anterior (TA) muscle mass of mice supplemented with 0%, 1%, or 1.5% SUC upon completion of the HIIT. Data are presented as mean ± SD. ^*^*P* < 0.05, ^**^*P* < 0.01, and ^***^*P* < 0.001 by one-way ANOVA with Bonferroni multiple comparisons; n = 10 per group.


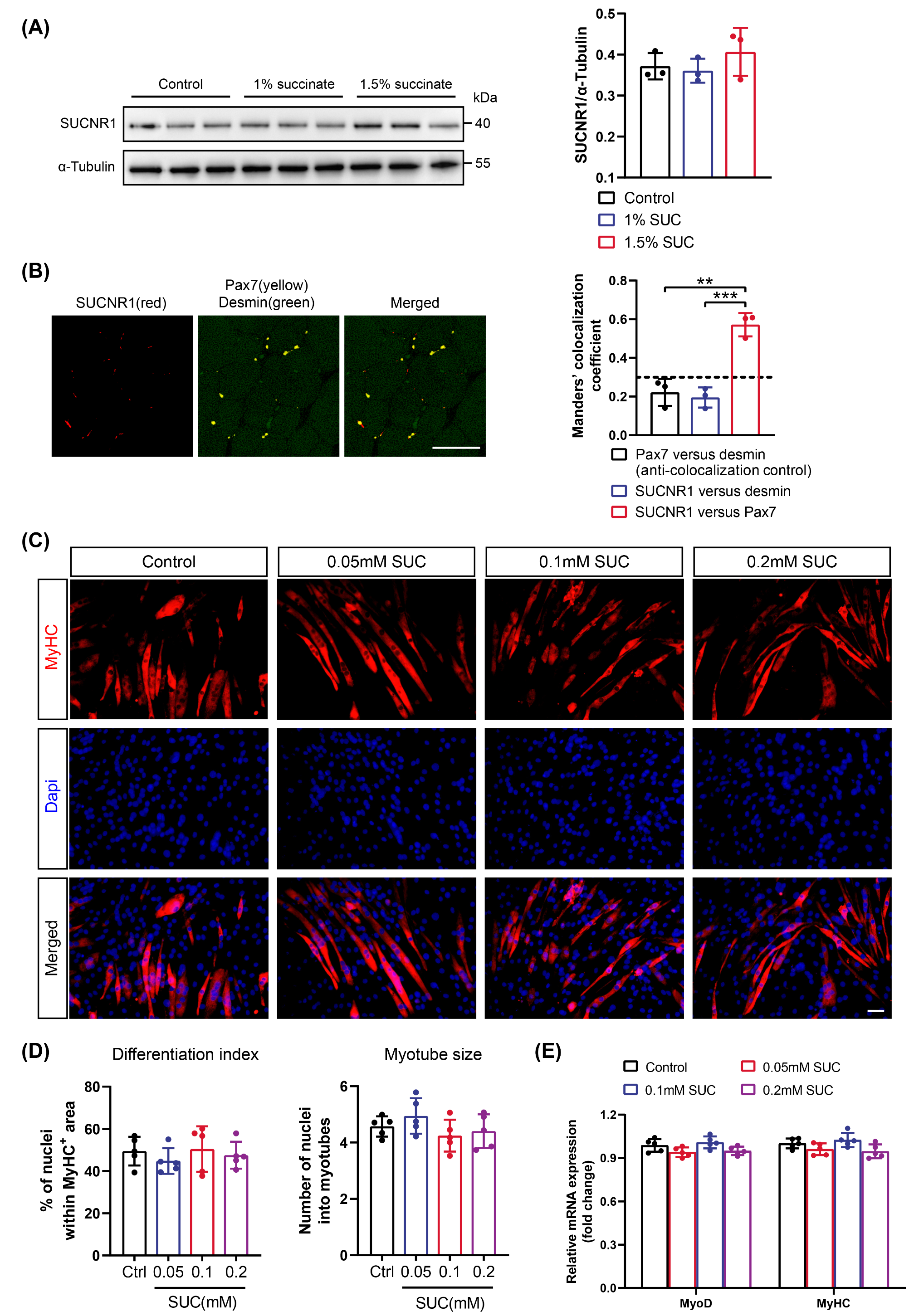


**Figure S2** The cellular localization of succinate receptor 1 (SUCNR1) in gastrocnemius (GA) muscle. (A) Western blotting and semi-quantitative analysis of SUCNR1 protein expression in GA muscle from mice supplemented with 0%, 1%, or 1.5% succinate (SUC). (B) Representative images of immunofluorescent staining for SUCNR1 (red), Pax7 (yellow), and desmin (green) in GA muscles of mice (left). Scale bars, 40 μm. Manders’ colocalization coefficient among SUCNR1, Pax7, and desmin (right). Coefficients of less than 0.3 are considered completely anti-colocalized, as validated by the anti-colocalization control (distinct cell type markers). (C) Representative images of immunofluorescence staining for myosin heavy chain (MyHC) (red) and DAPI (blue) in C2C12 myoblasts after SUC treatment during differentiation. Scale bars, 50 μm. (D) Quantification of differentiation index and myotube size in C2C12 myoblasts after SUC treatment during differentiation. (E) Real-time quantitative PCR analyses of *MyoD* and *MyHC* mRNA levels in C2C12 myoblasts after SUC treatment during differentiation. Data are presented as mean ± SD. ^*^*P* < 0.05, ^**^*P* < 0.01, and ^***^*P* < 0.001 by one-way ANOVA with Bonferroni multiple comparisons; n = 3 per group (A, B), n = 5 per group (C, D, and E).


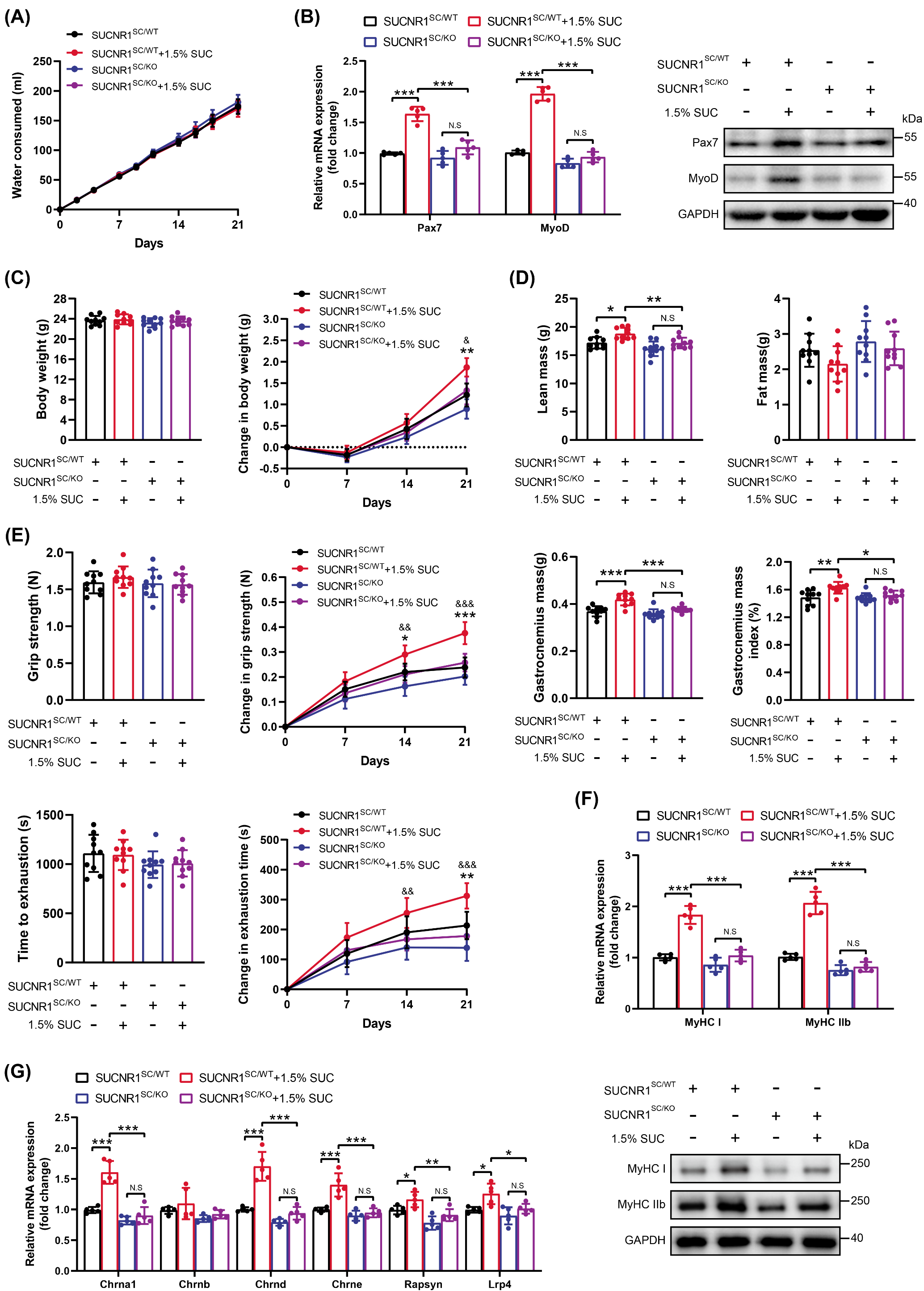


**Figure S3** Succinate receptor 1 (SUCNR1) in SCs mediates succinate-induced muscle adaption. (A) Water consumption during high-intensity interval training (HIIT). (B) Real-time quantitative PCR analyses of *PAX7* and *MyoD* mRNA levels in gastrocnemius (GA) muscles from SUCNR1^SC/WT^ and SUCNR1^SC/KO^ mice supplemented with or without 1.5% succinate (SUC) (left). PAX7 and MyoD protein expression as determined by western blotting in GA muscles from the four groups (right). (C) Baseline body weight (left) and absolute changes in body weight (right) from SUCNR1^SC/WT^ and SUCNR1^SC/KO^ mice supplemented with or without 1.5% SUC during HIIT. (D) Lean mass, fat mass, gastrocnemius mass, and gastrocnemius mass index from SUCNR1^SC/WT^ and SUCNR1^SC/KO^ mice supplemented with or without 1.5% SUC after completion of HIIT. (E) Baseline grip strength and exhaustion time from SUCNR1^SC/WT^ and SUCNR1^SC/KO^ mice (left). Absolute changes in grip strength and exhaustion time from SUCNR1^SC/WT^ and SUCNR1^SC/KO^ mice supplemented with or without 1.5% SUC during HIIT (right). (F) Real-time quantitative PCR analyses of myosin heavy chain (*MyHC*) *I* and *MyHC IIb* mRNA levels in GA muscles from SUCNR1^SC/WT^ and SUCNR1^SC/KO^ mice supplemented with or without 1.5% SUC (top). MyHC I and MyHC IIb protein expression as determined by western blotting in GA muscles from the four groups (bottom). (G) Real-time quantitative PCR analysis of *Chrna1*, *Chrnb*, *Chrnd*, *Chrne*, *Rapsyn*, and *Lrp4* mRNA levels in GA muscles from SUCNR1^SC/WT^ and SUCNR1^SC/KO^ mice supplemented with or without 1.5% SUC. Data are presented as mean ± SD. One-way ANOVA with Bonferroni multiple comparisons (BMC) was employed in A, B, C (left), D, E (left), F, and G. Two-way ANOVA with BMC was employed in C (right) and E (right) where ^*^SUCNR1^SC/WT^ + 1.5% SUC vs. SUCNR1^SC/WT^ and ^&^SUCNR1^SC/WT^ +1.5% SUC vs. SUCNR1^SC/KO^ +1.5% SUC. N.S., not significant, ^*^*P* or ^&^*P* < 0.05, ^**^*P* or ^&&^*P* < 0.01, and ^***^*P* or ^&&&^*P* < 0.001; n = 10 per group (A, C, D, and E), n = 5 per group [B (left), F (top), and G], n = 3 per group [B (right), F (bottom)].


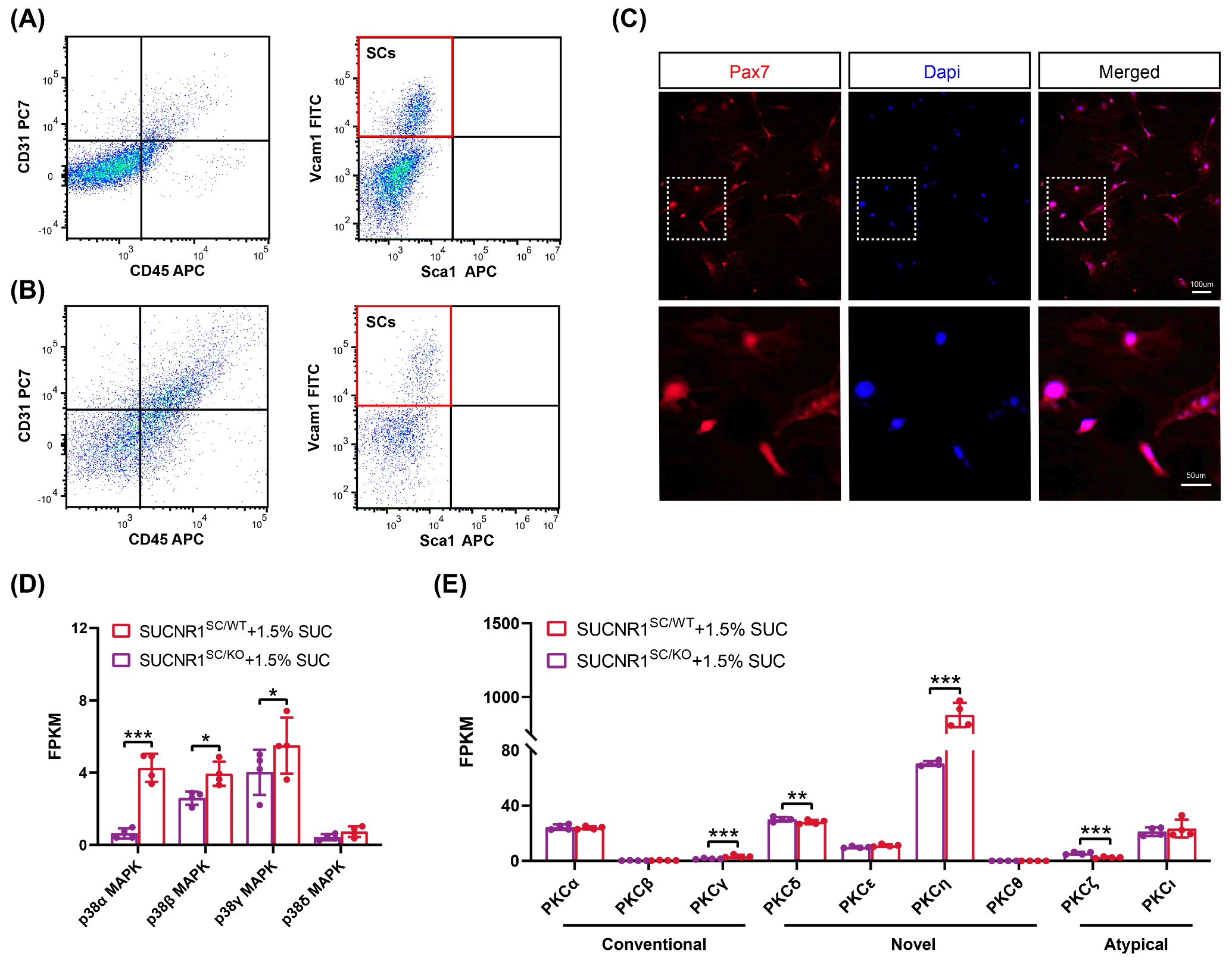


**Figure S4** Satellite cells (SCs) isolation and RNA sequencing. (A) Representative fluorescence activated cell sorting (FACS) plots of SCs isolated from SUCNR1^SC/WT^ mice supplemented with 1.5% succinate (SUC) after completing the high-intensity interval training (HIIT). (B) Representative FACS plots of SCs isolated from SUCNR1^SC/KO^ mice supplemented with 1.5% SUC after completing the HIIT. (C) Representative images of immunofluorescent staining for Pax7 (red) and DAPI (blue) in puriﬁed SC populations. (D) The gene expression levels of p38α, β, γ, and δ mitogen-activated protein kinase (MAPK) quantified based on the fragments per kilobase of transcript sequence per millions (FPKM) between SUCNR1^SC/WT^ and SUCNR1^SC/KO^ mice supplemented with 1.5% SUC. (E) Gene expression levels of conventional, novel, and atypical protein kinase C (PKC) isoforms quantified using the FPKM between SUCNR1^SC/WT^ and SUCNR1^SC/KO^ mice supplemented with 1.5% SUC. Negative binomial exact test with Benjamini–Hochberg correction was employed in D and E. ^*^*P* < 0.05, ^**^*P* < 0.01, and ^***^*P* < 0.001.


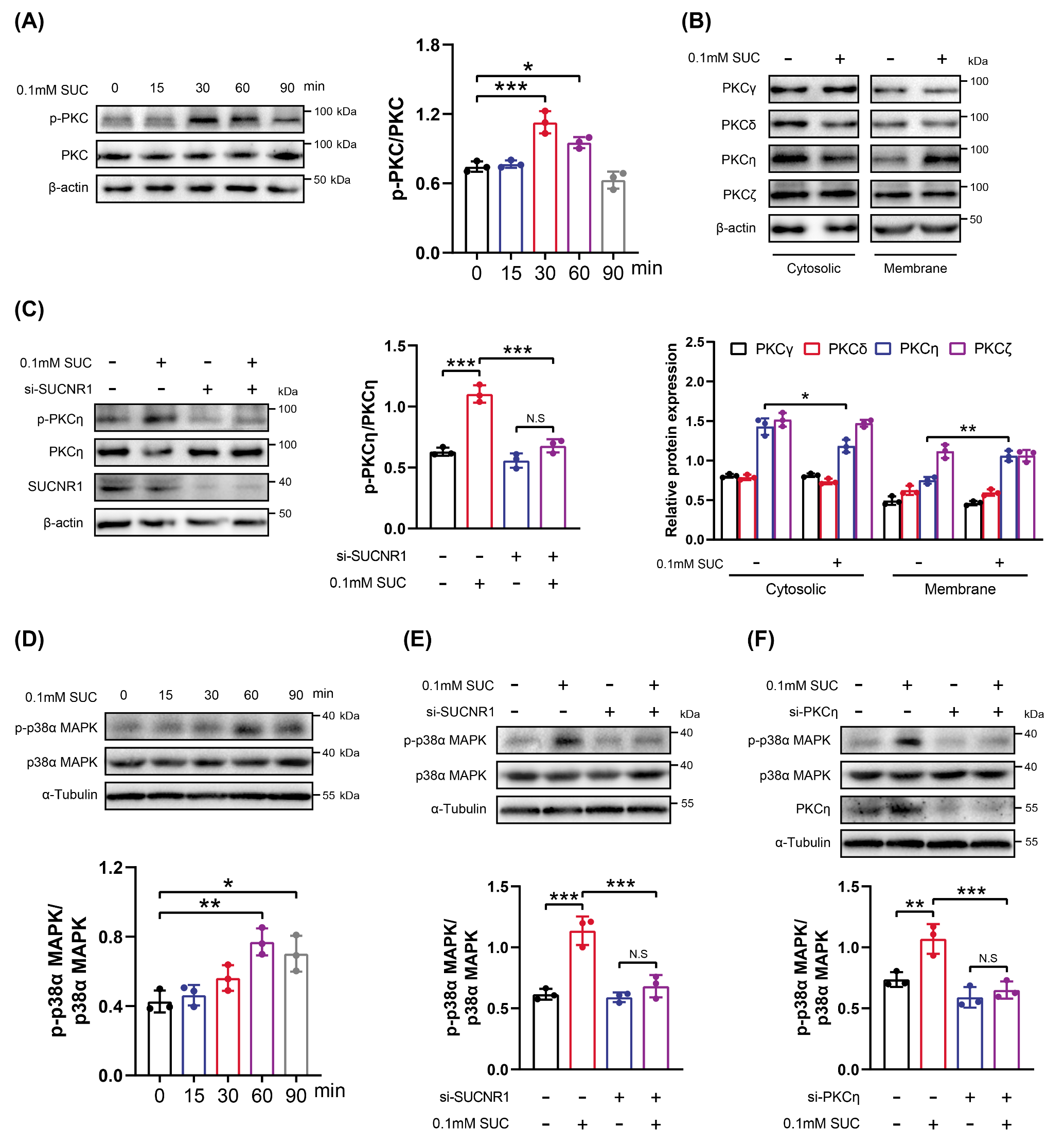


**Figure S5** Succinate activates the protein kinase Cη (PKCη)–p38α mitogen-activated protein kinase (MAPK) pathway through succinate receptor 1 (SUCNR1) in satellite cells (SCs). (A) Western blotting and semi-quantitative analyses of phosphorylated PKC (p-PKC) and PKC protein expression in isolated SCs treated with 0.1 mM succinate (SUC) for different time periods (0–90 min). (B) Western blotting and semi-quantitative analyses of PKC isoform protein expression in the membrane and cytoplasm of SCs treated with 0.1 mM SUC for 30 min. (C) Western blotting and semi-quantitative analyses of phosphorylated PKCη (p-PKCη), PKCη, and SUCNR1 protein expression in SCs transfected with SUCNR1 small interfering RNA (si-SUCNR1) and negative control (NC) after treatment with or without 0.1 mM SUC for 30 min. (D) Western blotting and semi-quantitative analyses of phosphorylated p38α MAPK (p-p38α MAPK) and p38α MAPK protein expression in isolated SCs treated with 0.1 mM SUC for different time periods (0–90 min). (E) Western blotting and semi-quantitative analyses of p-p38α MAPK and p38α MAPK protein expression in SCs transfected with si-SUCNR1 and NC after treatment with or without 0.1 mM SUC for 60 min. (F) Western blotting and semi-quantitative analyses of p-p38α MAPK, p38α MAPK, and PKCη protein expression in SCs transfected with si-PKCη and NC after treatment with or without 0.1 mM SUC for 60 min. Data are presented as mean ± SD. One-way ANOVA with Bonferroni multiple comparisons was employed in A, C, D, E, and F. Student's t-test was employed in B. ^*^*P* < 0.05, ^**^*P* < 0.01, and ^***^*P* < 0.001; n = 3 per group.


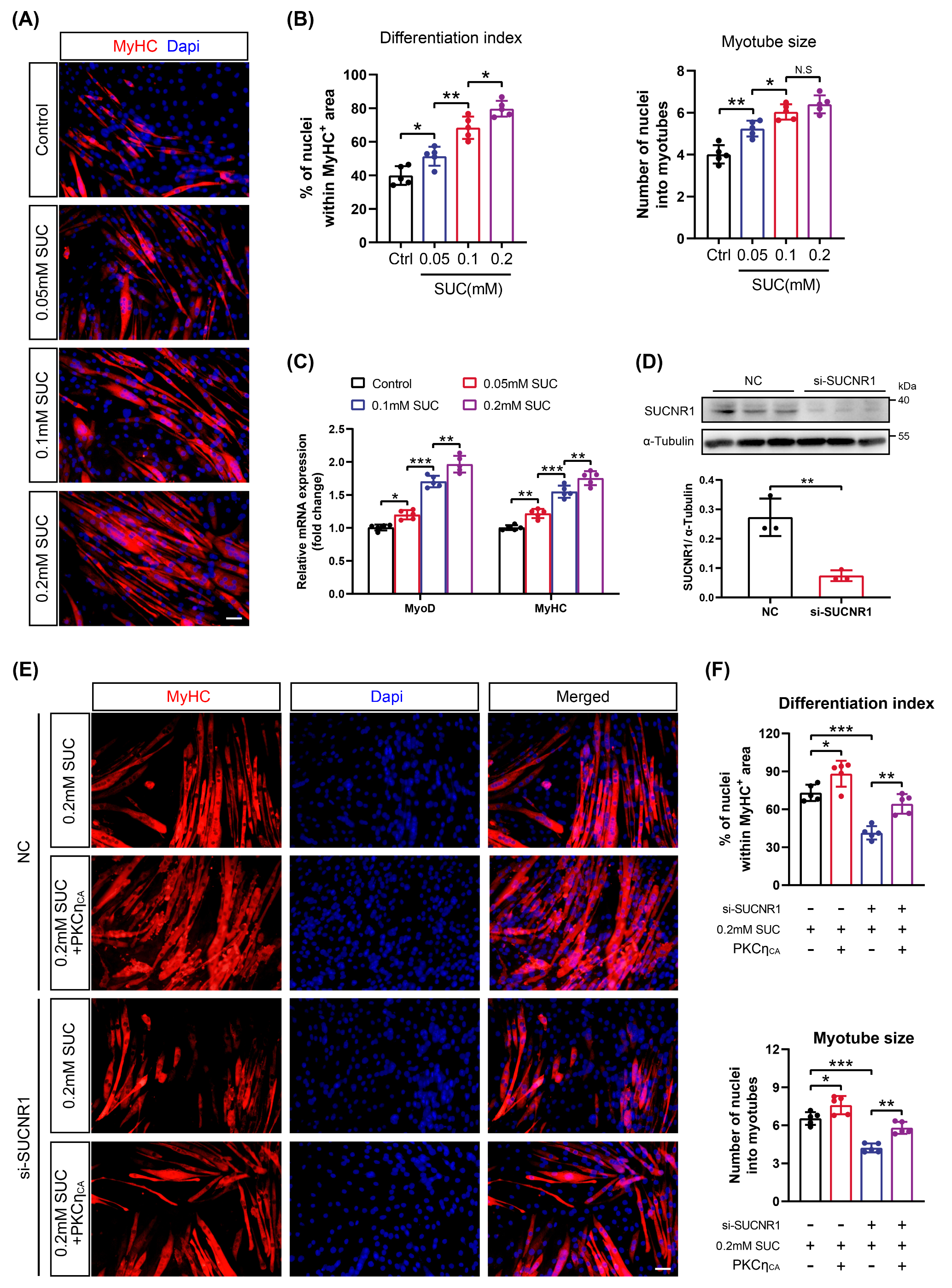


**Figure S6** Succinate enhances the myogenic differentiation of satellite cells (SCs) by succinate receptor 1 (SUCNR1)–protein kinase Cη (PKCη) activation. (A) Representative images of immunofluorescence staining for myosin heavy chain (MyHC) (red) and DAPI (blue) in isolated SCs following succinate (SUC) treatment during differentiation. Scale bars, 50 μm. (B) Quantification of differentiation index and myotube size in SCs following SUC treatment during differentiation. (C) Real-time quantitative PCR analyses of *MyoD* and *MyHC* mRNA levels in SCs following SUC treatment during differentiation. (D) Western blotting and semi-quantitative analysis of SUCNR1 protein expression in SCs transfected with SUCNR1 small interfering RNA (si-SUCNR1) and negative control (NC). (E) Representative images of immunofluorescence staining for MyHC (red) and DAPI (blue) in SCs co-transfected with empty plasmid (vector) or plasmid expressing constitutively active PKCη (PKCη_CA_) and si-SUCNR1 or NC after treatment with 0.2 mM SUC during differentiation. Scale bars, 50 μm. (F) Quantification of differentiation index and myotube size in SCs co-transfected with vector or PKCη_CA_ and si-SUCNR1 or NC after treatment with 0.2 mM SUC during differentiation. Data are presented as mean ± SD. One-way ANOVA with Bonferroni multiple comparisons was employed in B, C, and F. Student's t-test was employed in D. N.S., not significant, ^*^*P* < 0.05, ^**^*P* < 0.01, and ^***^*P* < 0.001; n = 5 per group (A, B, C, E, and F), n = 3 per group (D).


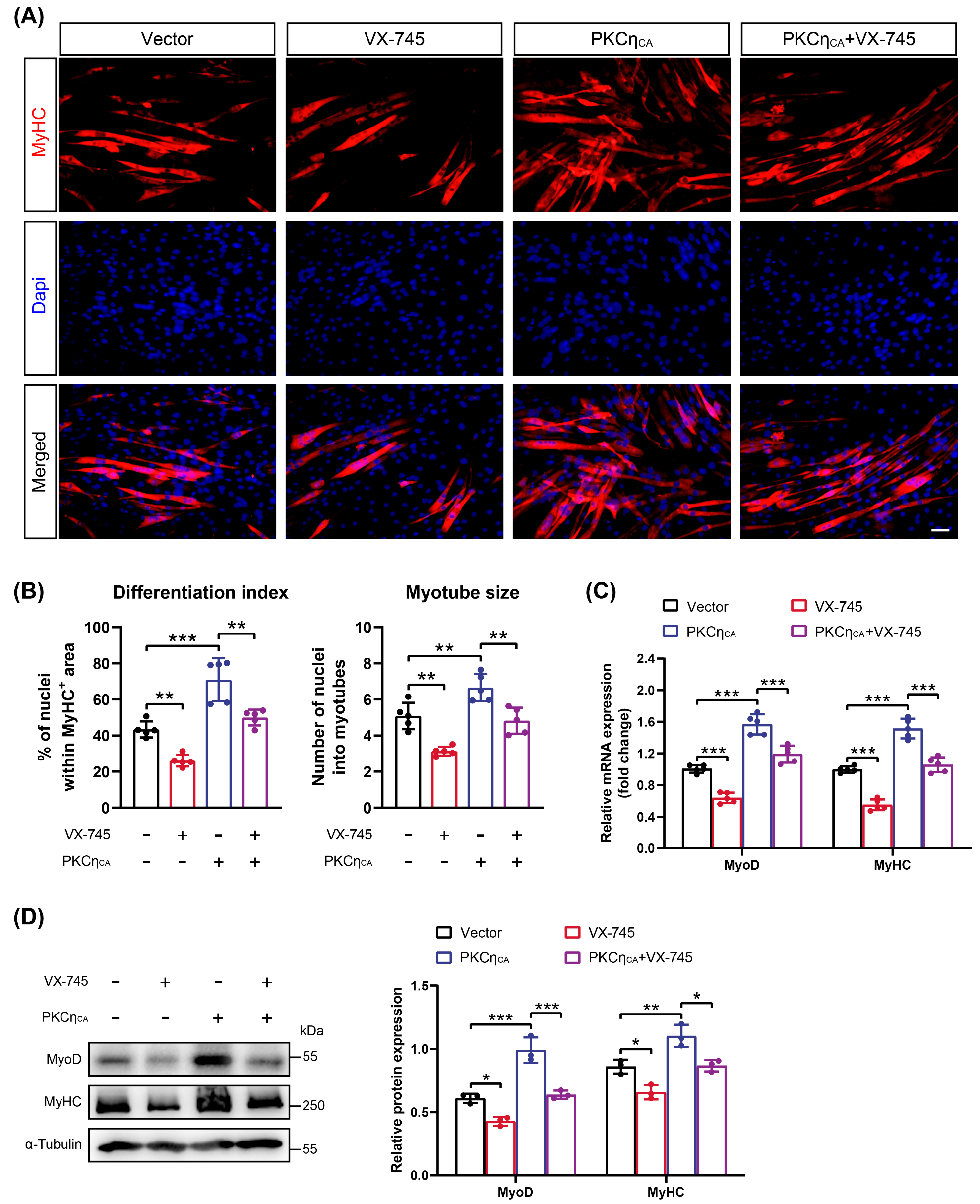


**Figure S7** The effects of p38α mitogen-activated protein kinase (MAPK) on protein kinase Cη (PKCη)-induced satellite cell (SC) differentiation. (A) Representative images of immunofluorescence staining for myosin heavy chain (MyHC) (red) and DAPI (blue) in SCs transfected with empty plasmid (vector) and plasmid expressing constitutively active PKCη (PKCη_CA_) after treatment with or without VX-745 during differentiation. Scale bars, 50 μm. (B) Quantification of differentiation index and myotube size in SCs transfected with vector and PKCη_CA_ after treatment with or without VX-745 during differentiation. (C) Real-time quantitative PCR analyses of *MyoD* and *MyHC* mRNA levels in SCs transfected with vector and PKCη_CA_ after treatment with or without VX-745 during differentiation. (D) Western blotting and semi-quantitative analyses of MyoD and MyHC protein expression in SCs transfected with vector and PKCη_CA_ after treatment with or without VX-745 during differentiation. Data are presented as mean ± SD. ^*^*P* < 0.05, ^**^*P* < 0.01, and ^***^*P* < 0.001 by one-way ANOVA with Bonferroni multiple comparisons; n = 5 per group (A, B, and C), n = 3 per group (D).


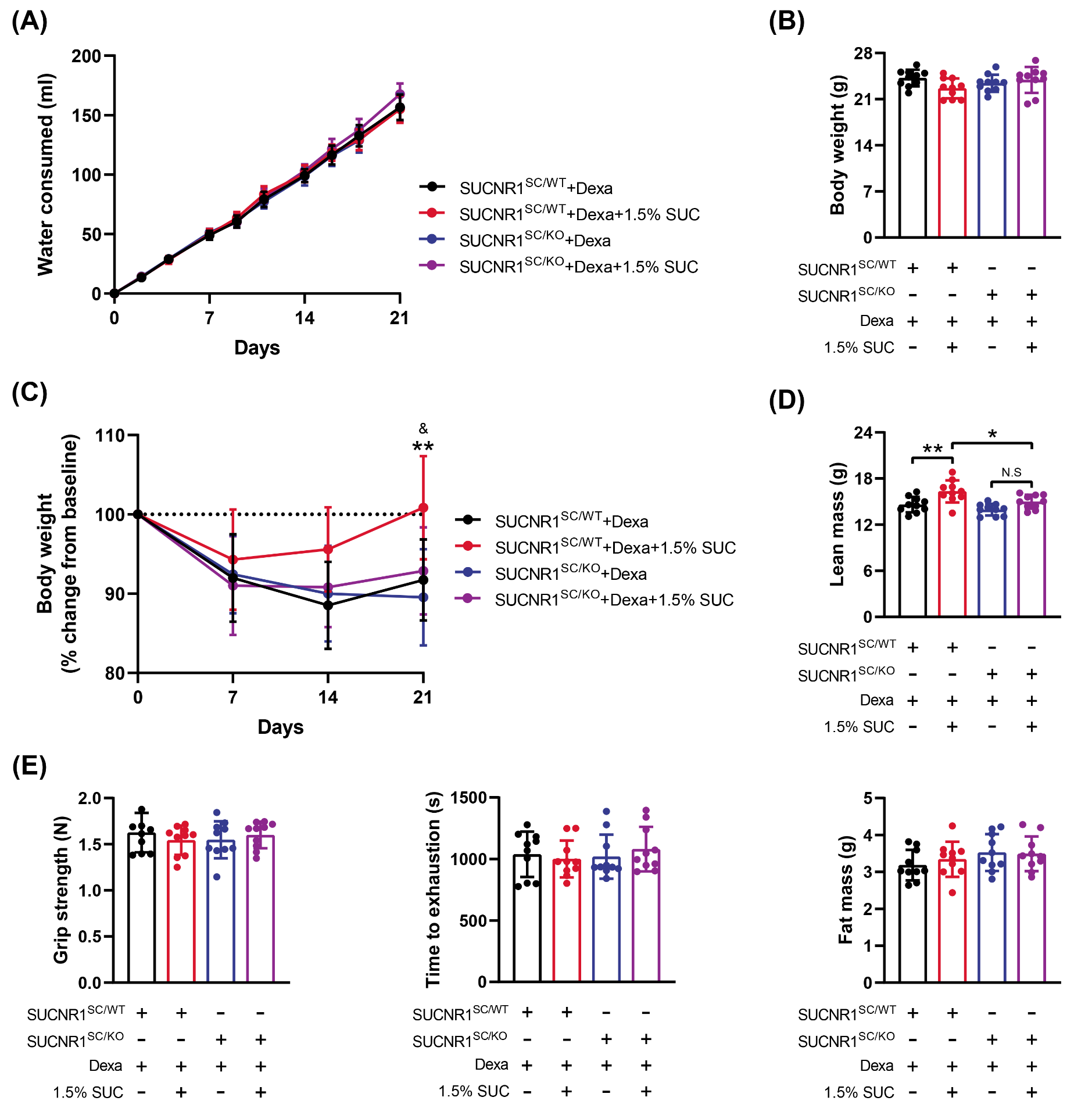


**Figure S8** The impact of succinate (SUC) on skeletal muscle in dexamethasone (Dexa)-treated mice. (A) Water consumption during Dexa treatment. (B) Baseline body weight of mice. (C) Percentage change from baseline of body weight in mice. (D) Lean mass and fat mass of SUCNR1^SC/WT^ and SUCNR1^SC/KO^ mice injected by Dexa and supplemented with or without 1.5% SUC. (E) Grip strength and exhaustion time of mice at baseline. Data are presented as mean ± SD. One-way ANOVA with Bonferroni multiple comparisons (BMC) was employed in A, B, D, and E. Two-way ANOVA with BMC was employed in C where ^*^SUCNR1^SC/WT^ + Dexa + 1.5% SUC vs. SUCNR1^SC/WT^ + Dexa and ^&^SUCNR1^SC/WT^ + Dexa + 1.5% SUC vs. SUCNR1^SC/KO^ + Dexa + 1.5% SUC. N.S., not significant, ^*^*P* or ^&^*P* < 0.05, ^**^*P* < 0.01; n = 10 per group.


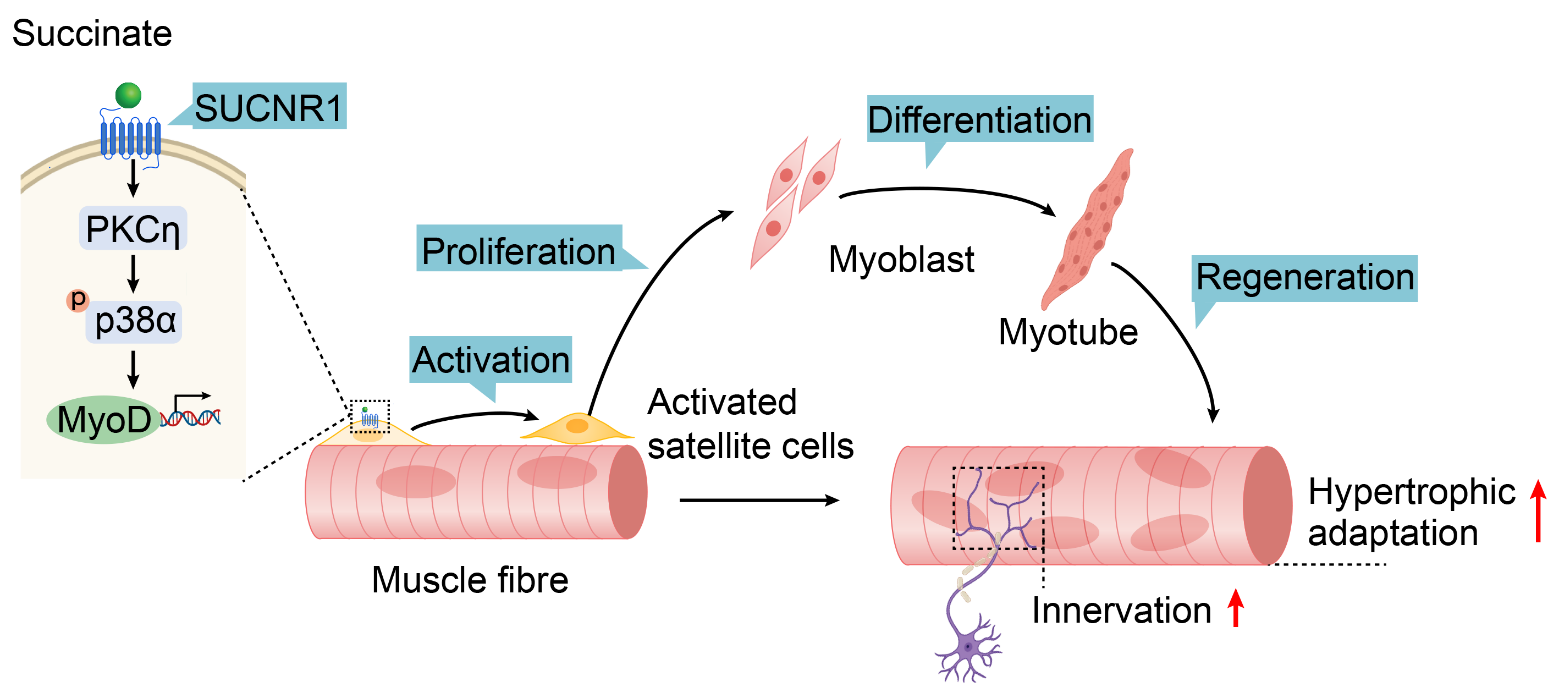


**Figure S9** The schematic diagram depicting succinate in modulating muscle adaptive remodeling. Succinate supplementation promotes the myogenic differentiation of satellite cells through activating SUCNR1–PKCη–p38α MAPK pathway, which drives muscle adaptive remodeling, including enhanced muscle hypertrophy and innervation.

**Supplemental Tables**

**Table S1. Antibodies used for immunofluorescence (IF) or western blotting (WB).**

| **Primary antibodies** | **Company** | **Cat.n** | **Use** | **Concentration** |
| --- | --- | --- | --- | --- |
| Laminin | Thermo Fisher | PA1-16730 | IF | 1:1000 |
| MyHC I | DSHB | BA-D5 | IF, WB | 1:10, 1:100 |
| MyHC IIa | DSHB | SC-71 | IF, WB | 1:20, 1:200 |
| MyHC IIb | DSHB | BF-F3 | IF, WB | 1:20, 1:200 |
| GAPDH | Abcam | ab8245 | WB | 1:1000 |
| Neurofilament | DSHB | 2H3 | IF | 1:50 |
| Synaptic vesicle protein 2 | DSHB | SV2 | IF | 1:50 |
| Pax7 | Abcam | ab187339 | IF, WB | 1:200, 1:1000 |
| Pax7 | DSHB | PAX7 | IF | 1:20 |
| MyoD | Santa Cruz | sc-377460 | IF, WB | 1:500, 1:1000 |
| SUCNR1 | Novus | NBP1-00861 | IF, WB | 1:200, 1:1000 |
| Desmin | Abcam | ab227651 | IF | 1:100 |
| MyHC | Santa Cruz | sc-376157 | IF, WB | 1:250, 1:1000 |
| p38α MAPK | Santa Cruz | sc-81621 | WB | 1:1000 |
| p-p38α MAPK | Thermo Fisher | MA5-15177 | WB | 1:1000 |
| PKC | Santa Cruz | sc-17769 | WB | 1:1000 |
| p-PKC | Abcam | ab109539 | WB | 1:2000 |
| PKCγ | Santa Cruz | sc-166385 | WB | 1:1000 |
| PKCδ | Santa Cruz | sc-8402 | WB | 1:1000 |
| PKCη | Abcam | ab179524 | WB | 1:1000 |
| p-PKCη | Abcam | ab5798 | WB | 1:1000 |
| PKCζ | Santa Cruz | sc-17781 | WB | 1:10000 |
| GFP | Santa Cruz | sc-9996 | WB | 1:1000 |
| β-actin | Proteintech Group | 20536-1-AP | WB | 1:5000 |
| α-tubulin | Abcam | ab7291 | WB | 1:5000 |

**Table S2. Primer sequences.**

| **Name** | **Forward Primer** | **Reverse Primer** |
| --- | --- | --- |
| MyHC I | CCCAGAAACAAGTGAAGAGCCT | GTTCCACGATGGCGATGTTC |
| MyHC IIa | TCAGGCTTCAGGATTTGGTGG | CTTGCGGAACTTGGATAGATTTG |
| MyHC IIb | AAGCCTGCCTCCTTCTTCATC | CTTAGCATCCACCACAAACACC |
| Myoglobin | TCGGTCTGTTTAAGACTCACCC | ATCTTGTGCTTGGTGGCGTGT |
| Tnnt1 | ACAGACTGGTCGGGAGATGAAAC | TGCGGTTGTAGAGCACATTGAT |
| Tnnt3 | GGGAACGCCAGAACAGATTG | ATCGCTAAGATGGTCAATGTTCAG |
| Chrna1 | CCACAGACTCAGGGGAGAAG | AACGGTGGTGTGTGTTGATG |
| Chrnb | GCTCTGAGGTCAGCCTGAAG | TCTCCCATTGGCCATTCTC |
| Chrne | GATTGGCATTGACTGGCACG | CCACTCCAAACTGCCCATC |
| Chrnd | GAGACCAAAACAGTTACAATGAGG | TGACGGGTGTCACCACAA |
| Rapsyn | ACGAGTGCGTGGAGGAGACT | TGTTCCTCTCCCCGATGGA |
| Lrp4 | GGCAAAAAGCAGGAACTTGT | TCTACCCAGTGGCCAGAACT |
| Pax7 | AGTATGGCCAAACTGCTGTTGAT | GTAGGCTTGTCCCGTTTCCA |
| MyoD | GACGGCTCTCTCTGCTCC | AAGTGTGCGTGCTCCTCC |
| MyHC | GAGTTCATTGACTTCGGGATGG | TGCTGCTCATACAGCTTGTTCTTG |
| Gapdh | CCTCGTCCCGTAGACAAAATG | TGAGGTCAATGAAGGGGTCGT |
